# Multi-tissue transcriptome-wide association study identifies novel candidate susceptibility genes for cataract

**DOI:** 10.3389/fopht.2024.1362350

**Published:** 2024-04-16

**Authors:** Hélène Choquet, Matthieu Duot, Victor A. Herrera, Sanjaya K. Shrestha, Travis J. Meyers, Thomas J. Hoffmann, Poorab K. Sangani, Salil A. Lachke

**Affiliations:** ^1^ Kaiser Permanente Northern California (KPNC), Division of Research, Oakland, CA, United States; ^2^ Department of Biological Sciences, University of Delaware, Newark, DE, United States; ^3^ The National Centre for Scientific Research (CNRS), IGDR (Institut de Génétique et Développement de Rennes) - Joint Research Units (UMR), Univ Rennes, Rennes, France; ^4^ Institute for Human Genetics, University of California San Francisco (UCSF), San Francisco, CA, United States; ^5^ Department of Epidemiology and Biostatistics, UCSF, San Francisco, CA, United States; ^6^ Department of Ophthalmology, KPNC, South San Francisco, CA, United States; ^7^ Center for Bioinformatics and Computational Biology, University of Delaware, Newark, DE, United States

**Keywords:** genetics, TWAS - transcriptome-wide association study, gene expression, lens tissue, cataract, multi-tissue analysis

## Abstract

**Introduction:**

Cataract is the leading cause of blindness among the elderly worldwide. Twin and family studies support an important role for genetic factors in cataract susceptibility with heritability estimates up to 58%. To date, 55 loci for cataract have been identified by genome-wide association studies (GWAS), however, much work remains to identify the causal genes. Here, we conducted a transcriptome-wide association study (TWAS) of cataract to prioritize causal genes and identify novel ones, and examine the impact of their expression.

**Methods:**

We performed tissue-specific and multi-tissue TWAS analyses to assess associations between imputed gene expression from 54 tissues (including 49 from the Genotype Tissue Expression (GTEx) Project v8) with cataract using FUSION software. Meta-analyzed GWAS summary statistics from 59,944 cataract cases and 478,571 controls, all of European ancestry and from two cohorts (GERA and UK Biobank) were used. We then examined the expression of the novel genes in the lens tissue using the iSyTE database.

**Results:**

Across tissue-specific and multi-tissue analyses, we identified 99 genes for which genetically predicted gene expression was associated with cataract after correcting for multiple testing. Of these 99 genes, 20 (*AC007773.*1, *ANKH*, *ASIP*, *ATP13A2*, *CAPZB*, *CEP95*, *COQ6*, *CREB1*, *CROCC*, *DDX5*, *EFEMP1*, *EIF2S2*, *ESRRB*, *GOSR2*, *HERC4*, *INSRR*, *NIPSNAP2*, *PICALM*, *SENP3*, and *SH3YL1*) did not overlap with previously reported cataract-associated loci. Tissue-specific analysis identified 202 significant gene-tissue associations for cataract, of which 166 (82.2%), representing 9 unique genes, were attributed to the previously reported 11q13.3 locus. Tissue-enrichment analysis revealed that gastrointestinal tissues represented one of the highest proportions of the Bonferroni-significant gene-tissue associations (21.3%). Moreover, this gastrointestinal tissue type was the only anatomical category significantly enriched in our results, after correcting for the number of tissue donors and imputable genes for each reference panel. Finally, most of the novel cataract genes (e.g., *Capzb*) were robustly expressed in iSyTE lens data.

**Discussion:**

Our results provide evidence of the utility of imputation-based TWAS approaches to characterize known GWAS risk loci and identify novel candidate genes that may increase our understanding of cataract etiology. Our findings also highlight the fact that expression of genes associated with cataract susceptibility is not necessarily restricted to lens tissue.

## Introduction

Cataract is the leading cause of blindness among older people worldwide and is a leading cause of vision loss in the United States (U.S.), affecting 22% of Americans aged 40 years and older (1). Cataracts are characterized by the opacification of the crystalline lens, leading to progressive loss of vision. Risk factors for cataract include type 2 diabetes, high blood pressure, high body mass index, myopic refractive error, cigarette smoking, and alcohol consumption (2). However, in a recent Mendelian randomization study, we demonstrated that only genetically determined myopic refractive error and primary open-angle glaucoma were significantly associated with cataract risk (3). In addition, women have a higher cataract burden than men of the same age (4), however it is not clear why this sex difference exists.

Twin and family studies strongly support an important role for genetic factors in cataract risk with heritability estimates up to 58% (5–10). Over the past few years, genome-wide association studies (GWASs) have identified more than 50 genetic susceptibility loci for cataracts in adults (11–13). Although those GWASs revealed many genetic loci associated with cataract susceptibility, the causal genes underlying those associations remain poorly understood. Moreover, the role of potential causal genes in the lens and other tissues and cataract is unknown.

We have previously conducted a multiethnic GWAS meta-analysis of cataract (11), using the Kaiser Permanente Northern California (KPNC) Genetic Epidemiology Research on Adult Health and Aging (GERA) cohort, the UK Biobank, and data from the 23andMe research cohort, and identified 55 genetic loci associated at a genome-wide level of significance (*P* < 5 × 10^–8^) with cataract (11). Interestingly, one of these loci (*CASP7*) was specific to women (11). However, the number of risk factors associated with cataract specifically in women that may explain the sex difference in disease burden remain limited.

Recently, transcriptome-wide association study (TWAS) approaches have been developed to characterize established GWAS risk loci and uncover additional gene–disease associations (14–19). These TWAS approaches leverage data from GWAS and expression quantitative trait loci (eQTL) to impute differential expression and test for gene expression associated with the GWAS disease of interest. TWASs have been fruitful in detecting functioning genes regulated by disease-associated variants, thus providing important insight into mechanisms of diseases (19).

In addition to GWAS findings, our previous GWAS meta-analysis of cataract (11) also reported positive genetic correlations between cataract with disorders other than eye disorders, including chronic pulmonary and gastrointestinal diseases. For this reason, we hypothesized that tissues in other anatomical parts of the body than the eye could be relevant to investigate, to better understand the mechanisms underlying cataract.

In this study, we conducted a TWAS of cataract to identify novel associated genes and interpret the transcriptional and disease risk mechanisms for cataract susceptibility genes. We imputed gene expression into GWAS data (59,944 cataract cases and 478,571 controls of European ancestry from GERA and UK Biobank cohorts) from our previous GWAS (11) using eQTL datasets (20) from multiple tissues (54 tissue reference panels). We conducted tissue-specific and multi-tissue TWAS analyses, as well as tissue type-enrichment analysis. Finally, we subsequently fine-mapped those associations and examined the expression of the novel genes identified in the current TWAS in lens tissues using the iSyTE database (21–24). The data sources used for the current TWAS study and TWAS analyses and results are summarized in a flowchart (**Supplementary Figure S1**).

## Methods

### Cataract GWAS data

We used summary statistics from our recent GWAS meta-analysis (11). Briefly, we conducted a meta-analysis, including 538,515 individuals of European ancestry (59,944 cataract cases and 478,571 controls) from the GERA (25) and UK Biobank (26, 27) cohorts. The meta-analysis was performed using the R package “meta” (28) and fixed-effects summary estimates were calculated for an additive model. In total, 9,056,148 single nucleotide variations (SNVs) passing quality control were used for the TWAS analyses.

For the GERA cohort, all study procedures were approved by the Institutional Review Board of the Kaiser Permanente Northern California, and written informed consent was obtained from all participants. For the UK Biobank, this research has been conducted using the UK Biobank Resource project #14105.

### FUSION eQTL data

Local (*cis*) eQTL datasets for 54 tissue types were downloaded from the FUSION website. These reference data were sourced from the Genotype Tissue Expression (GTEx) Project v8 (N=49 tissue reference panels) (20), the CommonMind Consortium (CMC) (N=2 tissue reference panels) (29), the Metabolic Syndrome in Men Study (METSIM) (N=1 tissue reference panel) (30), the Netherlands Twin Registry (NTR) (N=1 tissue reference panel) (31), and the Cardiovascular Risk in Young Finns Study (YFS) (N=1 tissue reference panel) (32). **Supplementary Table S1** reports the datasets sources, number of individuals, and number of imputable genes for each tissue reference panel.

### Tissue-specific TWAS analyses

We conducted a TWAS of cataract using FUSION (18), which computes predictive models for eQTLs from reference data, and tests the association between predicted gene expression with a trait from GWAS summary statistics. As previously done (33), we performed tissue-specific TWAS analyses using FUSION default settings and the three following data inputs: 1) the above-mentioned GWAS summary statistics for cataract; 2) FUSION gene expression predictive models for 54 reference tissues; and 3) 1000 Genomes (European ancestry) Phase 3 data from the 1000 Genomes Project (34) as a reference panel for linkage disequilibrium (LD). Model weights for tissue-specific gene expression regressed on SNVs were computed from best linear unbiased predictor (BLUP), Bayesian sparse linear mixed model (BSLMM), least absolute shrinkage and selection operator (LASSO), and elastic net regression, as well as from the model with the top associated SNV.

A total of 325,513 gene-tissue-pairs (representing 37,920 unique genes across 54 tissue reference panels) were tested for associations between imputed gene expression with cataract susceptibility. Associations with a Bonferroni significance p-value less than 1.54 x 10^-7^ (=0.05/325,513) were considered significant. Novel TWAS genes were defined as those located over 1 Mb apart from any previously reported cataract GWAS loci (i.e., no prior GWAS SNVs within 1 Mb from the start or end of the gene).

### Colocalization analyses

To assess whether GWAS SNVs colocalized with eQTLs, we conducted a Bayesian colocalization analysis using the COLOCv3.2.1 software, which is implemented in FUSION using marginal expression weights, for Bonferroni-significant TWAS associations (35). Thus, we tested the hypothesis that a single variant in each TWAS-significant model was associated with both cataract (from the GWAS) and imputed gene expression. Bayesian posterior probability greater than 0.9 was considered supporting evidence for colocalization.

### Conditional and joint analyses

To determine if the TWAS associations were conditionally independent of the GWAS hits, we conducted conditional analyses by adjusting transcriptome-wide associations for SNV-level effects from GWAS. Specifically, we used the COJO software program to adjust the GWAS summary statistics (the meta-analyzed results from the GERA and UK Biobank European samples) by the most statistically significant risk variants within 1 Mb of each TWAS gene (36). Using the marginal TWAS associations from the single-tissue analysis, we performed a FUSION joint analysis for cataract-associated genes located on the same chromosome region within each reference panel.

### Tissue enrichment analyses

To identify tissues potentially relevant to cataract, we assigned the 54 tissue reference panels to 12 anatomical categories as per Strunz et al. (2020) (37): adipose (n = 3 reference panels), brain (n = 15), cardiovascular (n = 9), female reproductive (n = 3), gastrointestinal (n = 7), gland (n = 11), lung (n = 1), skeletal muscle (n = 1), skin (n = 2), tibial nerve (n = 1), and transformed fibroblasts (n = 1). **Supplementary Table S1** lists the tissue reference panels and their corresponding anatomical categories used for this analysis. We assessed the frequency of Bonferroni-significant TWAS genes in each anatomical category. Because more Bonferroni-significant TWAS genes are expected from eQTL reference panels with more tissue donors and more imputable genes, we used the hypergeometric test to estimate the probability of observing at least as many TWAS-significant genes from all the gene-tissue pairs that we tested in each anatomical category.

### Sex-specific TWAS analyses

We also conducted sex-specific TWAS analyses using sex-specific GWAS summary statistics (i.e., women and men analyzed separately) and tissue reference panels (i.e., ovary, uterus, and vagina eQTLs for women; and prostate and testis eQTLs for men).

### Multi-tissue TWAS analysis

We conducted an omnibus test in FUSION for associations with cataract across multiple tissues. Specifically, TWAS associations from all 54 tissue reference panels were jointly analyzed accounting for correlation between expression weights across tissues. Two filters were applied to the omnibus test results to consider a multi-tissue gene expression test significant: 1) using a Bonferroni correction, we divided the α of 0.05 by the effective number of genes tested (n = 13,328), and retained genes with omnibus test p-values less than this value (P < 3.75x10^-6^); and 2) genes with a minimum tissue-specific p-value suggestive of a significant association (P < 1x10^-5^) were retained as described by Barbeira et al (17).

### Expression of novel cataract-associated genes in lens tissues

The iSyTE 2.0 database, which contains meta-analyzed mouse lens gene expression data across different stages, was used to examine the expression of the novel genes identified in the current study in the lens tissue (21–23). Mouse orthologs of the human candidate genes for the novel cataract genes identified in the current TWAS analyses were examined in iSyTE, which contains meta-analyzed lens transcriptome data generated on microarrays or RNA-sequencing (RNA-seq) (21, 23). Mouse whole lens tissue gene expression datasets at embryonic day (E) stages E10.5, E11.5, E12.5, E16.5, E17.5, E19.5, and postnatal (P) day stages P0, P2, and P56, in addition to isolated lens epithelium at P28 were available on the Affymetrix 430 2.0 platform (GeneChip Mouse Genome 430 2.0 Array and/or 430A 2.0 Array) and were used in this analysis. Further, mouse whole lens tissue gene expression datasets at stages P4, P8, P12, P20, P30, P42, P52, and P60 were available on the Illumina platform (BeadChip MouseWG-6 v2.0 Expression arrays), and were used in this analysis. We also examined RNA-seq data generated on mouse whole lens tissue at E10.5, E12.5, E14.5 and E16.5. Additionally, because lens-enriched expression of a candidate gene has proven to be an effective predictor of its role in the lens (21, 22), the lens-enrichment of these candidate genes was also investigated at these stages. “Lens-enriched expression” is a measure of expression of a candidate gene in the lens compared to that in mouse whole embryonic body (WB) as described (21–23, 38, 39). Microarray expression data is publicly available on several gene-specific perturbation mouse models that exhibit lens defects or cataract, as described (21). We analyzed these datasets to examine potential changes in expression of the novel cataract candidate genes, as done before (11). Additionally, to gain insights into expression of candidate genes specifically in lens epithelial or fiber cells, we examined previously described RNA-seq data from isolated epithelium and fiber cells (40, 41). Gene expression analysis was performed as previously described (11, 23, 42). The University of Delaware Institutional Animal Care and Use Committee (IACUC) reviewed and approved the animal protocol.

## Results

### Tissue-specific TWAS analysis identified 202 gene-tissue pairs associated with cataract

**Figure 1:**
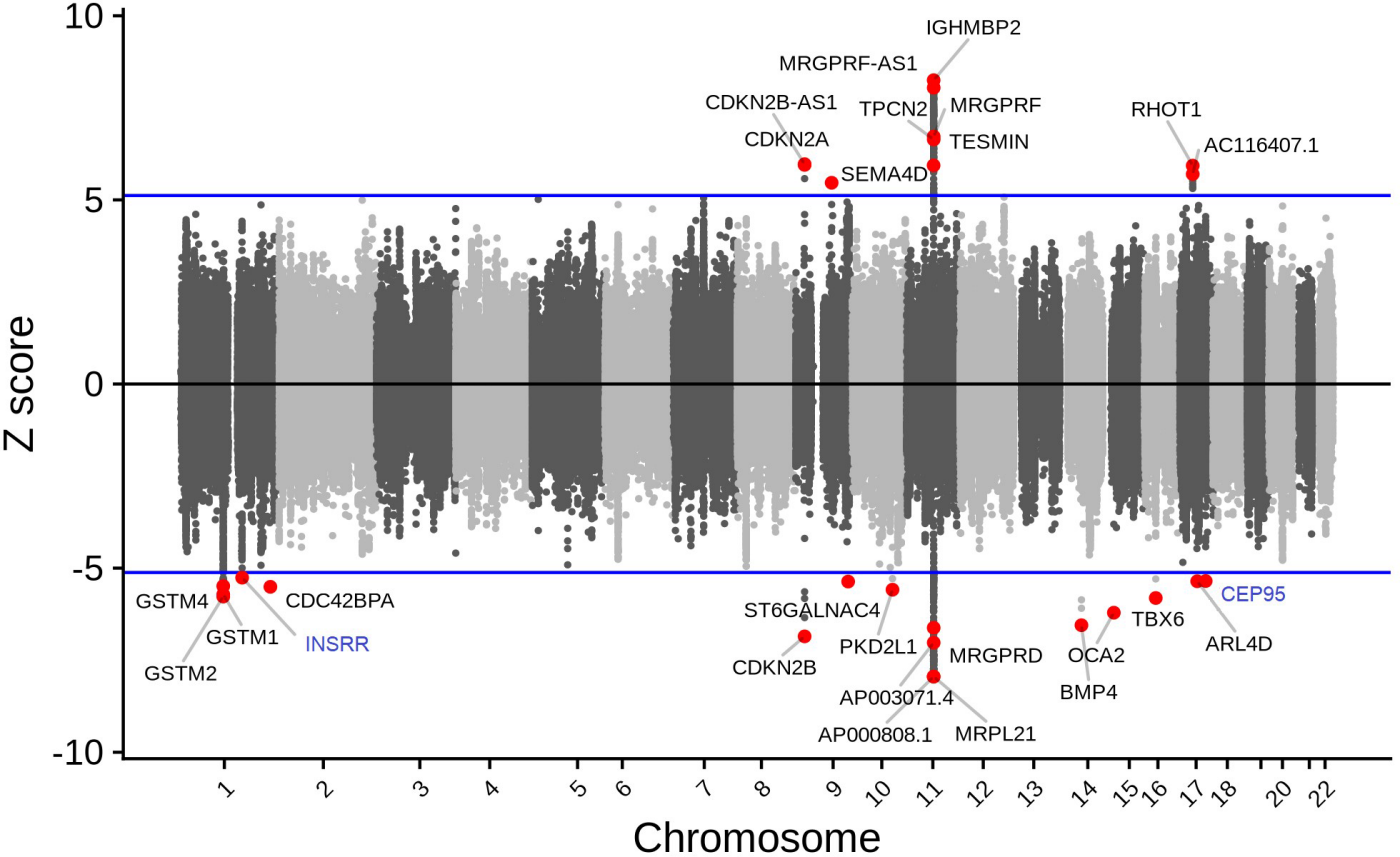
Tissue-specific TWAS analysis identified 27 unique genes associated with cataract. While increased predicted expression was associated with cataract risk for 10 genes (i.e., genes with z > 5.0; which corresponds to the results presented on the upper panel), decreased predicted expression was associated with cataract risk for 17 genes (i.e., genes with z < -5.0; which corresponds to the results presented on the lower panel). Genes in blue are novel (i.e., no prior reported GWAS SNV within 1 Mb).

**Figure 2:**
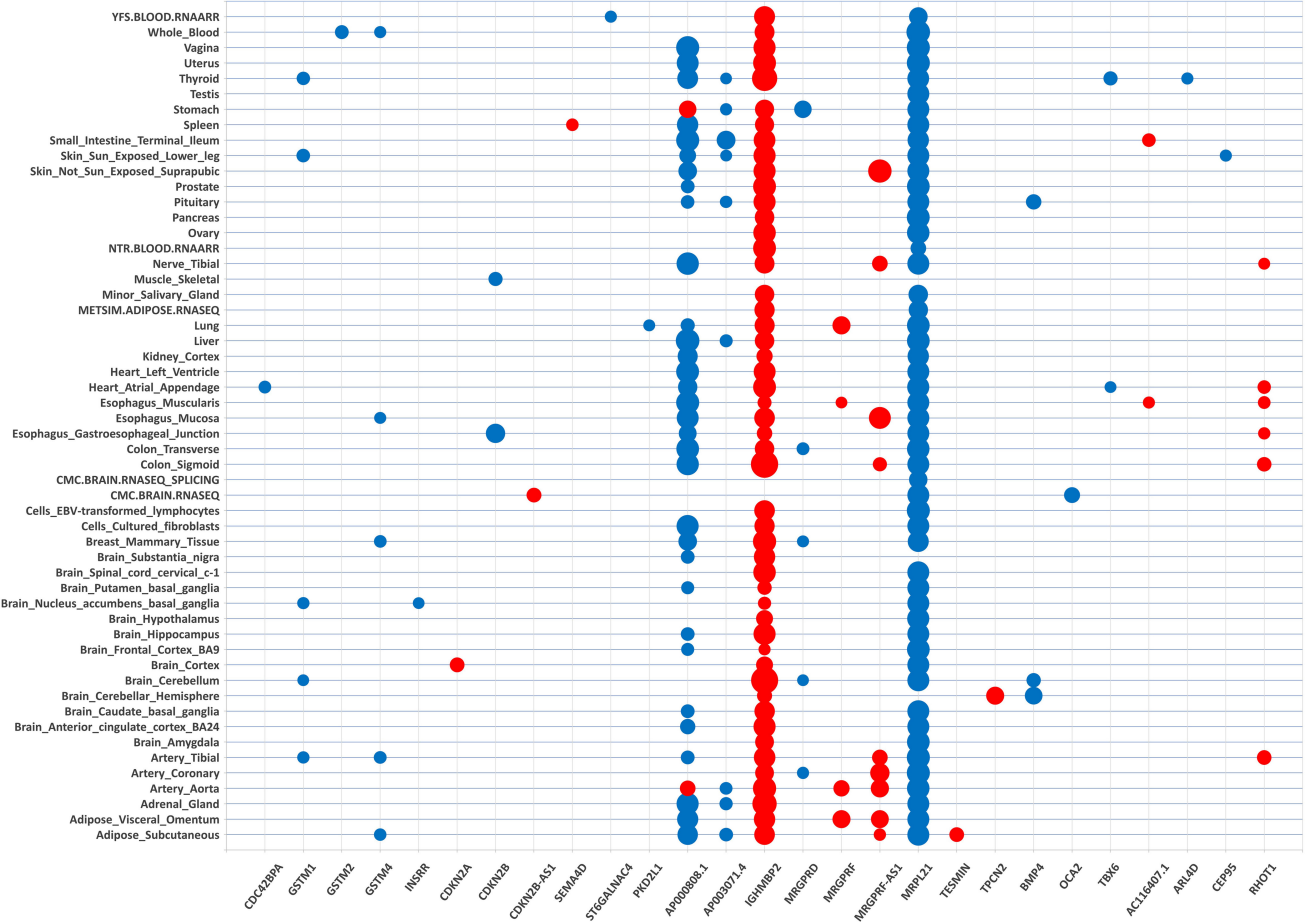
Transcriptome-wide association matrix of cataract significant gene-tissue associations. Tissue-specific TWAS analysis identified 202 gene-tissue pairs represented by 27 unique genes (on the x-axis) across 54 tissue reference panels (on the y-axis). The 27 unique genes are listed by chromosome position order (chr 1 on the left side of the matrix; chr 22 on the right side of the matrix). The tissue reference panels are listed by alphabetic order. The size of the dot for each gene-tissue association is proportional -log_10_ (TWAS.P). Color corresponds to the predicted direction of expression changes: red and blue for increased and decreased expression changes, respectively.

We found that 202 gene-tissue pairs reached the Bonferroni significance threshold for their associations between imputed gene expression with cataract susceptibility (**Supplementary Table S2**). While increased predicted expression was associated with cataract risk for 79 of the Bonferroni-significant gene-tissue pairs (e.g., *IGHMBP2*-colon sigmoid, z = 8.25), decreased predicted expression was associated with cataract for 123 Bonferroni-significant gene-tissue pairs (e.g., *MRPL21* - whole blood, z = -7.64) (**Figures 1**, **2**).

These 202 gene-tissue pairs were represented by 27 unique genes across 54 tissue reference panels (**Figures 1**, **2**). Importantly, 2 of the 27 unique genes did not overlap previously identified cataract GWAS loci: *INSRR* on chromosome 1, and *CEP95* on chromosome 17 (**Table 1**). Furthermore, out of the 27 genes, we found that 9 (33.3%) were located in the 11q13.3 genomic region which was previously identified in our GWAS (11). These include: *TESMIN*, *AP000808.1*, *MRPL21*, *IGHMBP2*, *MRGPRD*, *MRGPRF*, *AP003071.4*, *MRGPRF-AS1*, and *TPCN2* (**Figure 1**).

**Table 1:** TWAS analyses of cataract identified 99 unique genes.

GENE	Chr:position (GRCh37/hg19)	TWAS Type analyses	Novel gene (compared to known GWAS loci that reached GW level of significance)
***CROCC***	**chr1:17248426-17299459**	Multi-tissue TWAS	**novel**
***ATP13A2***	**chr1:17312453-17338423**	Multi-tissue TWAS	**novel**
***CAPZB***	**chr1:19665269-19810789**	Multi-tissue TWAS	**novel**
*GSTM4*	chr1:110198721-110204322	Tissue-specific TWAS	*GSTM2 -* PMID: 34127677
*GSTM2*	chr1:110210679-110217908	Tissue-specific TWAS	
*GSTM1*	chr1:110230439-110236367	Tissue-specific TWAS	
*ADAM15*	chr1:155023792-155031159	Multi-tissue TWAS	*DPM3-KRTCAP2 -* PMID: 34127677
*EFNA1*	chr1:155100352-155107375	Multi-tissue TWAS	
***INSRR***	**chr1:156809855-156828909**	Tissue-specific TWAS	**novel**
*ADCK3*	chr1:227127938-227175246	Multi-tissue TWAS	*ADCK3 -* PMID: 34127677
*CDC42BPA*	chr1:227177559-227506193	both	
***SH3YL1***	**chr2:218136-261130**	Multi-tissue TWAS	**novel**
*TRIB2*	chr2:12857062-12882860	Multi-tissue TWAS	near *TRIB2 -* PMID: 34127677
*TRMT61B*	chr2:29072687-29093175	Multi-tissue TWAS	*PLB1 -* PMID: 34127677
***EFEMP1***	**chr2:56093102-56150917**	Multi-tissue TWAS	**novel**
*CREB1*	chr2:208394616-208470284	Multi-tissue TWAS	**novel**
*DIRC3*	chr2:218148746-218621316	Multi-tissue TWAS	*DIRC3 -* PMID: 34127677
*CXCR2*	chr2:218990736-219001976	Multi-tissue TWAS	
*PPM1M*	chr3: 52279808-52284615	Multi-tissue TWAS	*NT5DC2 -* PMID: 34127677
*GLYCTK*	chr3:52321844-52329273	Multi-tissue TWAS	
*WDR82*	chr3:52288438-52312659	Multi-tissue TWAS	
*SEMA3G*	chr3:52467268-52479043	Multi-tissue TWAS	
*NT5DC2*	chr3:52558403-52567873	Multi-tissue TWAS	
*PBRM1*	chr3:52579383-52719615	Multi-tissue TWAS	
*GNL3*	chr3:52719936-52728513	Multi-tissue TWAS	
*SPCS1*	chr3:52739857-52742197	Multi-tissue TWAS	
*NEK4*	chr3:52742460-52804956	Multi-tissue TWAS	
*ITIH1*	chr3:52811615-52826078	Multi-tissue TWAS	
*PRKCD*	chr3:53195225-53226733	Multi-tissue TWAS	
*THOC7*	chr3:63819546-63849481	Multi-tissue TWAS	*ATXN7 -* PMID: 34127677
*ATXN7*	chr3:63884075-63989136	Multi-tissue TWAS	
***ANKH***	chr5:14704909-14871887	Multi-tissue TWAS	**novel**
*HLA-DQB1*	chr6:32627244-32634434	Multi-tissue TWAS	*3’ HLA-DQB1 -* PMID: 31816047
*QKI*	chr6:163835675-163999628	Multi-tissue TWAS	*QKI -* PMID: 34127677
*IGFBP3*	chr7:45951844-45960871	Multi-tissue TWAS	*IGFBP3-TNS3 -* PMID: 34127677
***NIPSNAP2***	**chr7:56032278-56067872**	Multi-tissue TWAS	**novel**
*C8orf58*	chr8:22457112-22461655	Multi-tissue TWAS	*BIN3-EGR3 -* PMID: 34127677
*BIN3*	chr8:22477931-22526634	Multi-tissue TWAS	
*CCAR2*	chr8:22462270-22477984	Multi-tissue TWAS	
*CDKN2B-AS1*	chr9:21994790-22077889	Tissue-specific TWAS	*CDKN2B-DMRTA1 -* PMID: 34127677
*CDKN2A*	chr9:21967751-21974856	Tissue-specific TWAS	
*CDKN2B*	chr9:22002902-22009304	Tissue-specific TWAS	
*DMRTA1*	chr9:22446823-22455739	Multi-tissue TWAS	
*SEMA4D*	chr9:91975702-92094805	both	*SEMA4D -* PMID: 34127677
*FKTN*	chr9:108320411-108403399	Multi-tissue TWAS	*FKTN-TAL2 -* PMID: 34127677
*ST6GALNAC4*	chr9:130670165-130679320	Tissue-specific TWAS	*ST6GALNAC4-PIP5KL1 -* PMID: 34127677
***HERC4***	chr10:69681656-69835103	Multi-tissue TWAS	**novel**
*PLCE1*	chr10:95753688-96092580	Multi-tissue TWAS	*PLCE1 -* PMID: 34127677
*NOC3L*	chr10:96092988-96122683	Multi-tissue TWAS	
*ABCC2*	chr10:101542397-101612351	Multi-tissue TWAS	*DNMBP -* PMID: 34127677
*PKD2L1*	chr10:102047906-102089985	both	
*ENTPD7*	chr10:101419266-101470998	Multi-tissue TWAS	
*CUTC*	chr10:101491991-101515891	Multi-tissue TWAS	
*DNMBP*	chr10:101635328-101673849	Multi-tissue TWAS	
*MRPL21*	chr11:68658746-68671300	both	11q13.3 *-* PMID: 34127677
*IGHMBP2*	chr11:68671359-68708069	both	
*TESMIN*	chr11:68474908-68518988	both	
*AP000808.1*	chr11:68708971-68710320	Tissue-specific TWAS	
*MRGPRD*	chr11:68747490-68748455	Tissue-specific TWAS	
*AP003071.4*	chr11:68768233-68769516	Tissue-specific TWAS	
*MRGPRF-AS1*	chr11:68779822-68785915	Tissue-specific TWAS	
*MRGPRF*	chr11:68771866-68780714	both	
*TPCN2*	chr11:68816400-68858065	both	
***PICALM***	**chr11:85668218-85780126**	Multi-tissue TWAS	**novel**
*CAPRIN2*	chr12:30862487-30907885	Multi-tissue TWAS	*CAPRIN2 -* PMID: 34127677
*UBE3B*	chr12:109915439-109928527	Multi-tissue TWAS	*MVK-FAM222A -* PMID: 34127677
*BMP4*	chr14:54416454-54420113	both	*BMP4 -* PMID: 34127677
***COQ6***	**chr14:74416629-74430373**	Multi-tissue TWAS	**novel**
***ESRRB***	**chr14:76837614-76968180**	Multi-tissue TWAS	**novel**
*OCA2*	chr15:28000021-28344461	both	*OCA2 -* PMID: 34127677
*MVP*	chr16:29831715-29859360	Multi-tissue TWAS	*ALDOA -* PMID: 34127677
*TBX6*	chr16:30097114-30103245	Tissue-specific TWAS	
*NFAT5*	chr16:69599869-69738569	Multi-tissue TWAS	*WWP2 -* PMID: 34127677
*NOB1*	chr16:69775757-69788871	Multi-tissue TWAS	
*WWP2*	chr16:69796186-69975644	Multi-tissue TWAS	
*CLEC18A*	chr16:69984805-69997889	Multi-tissue TWAS	
*NPIPB14P*	chr16:70010291-70030091	Multi-tissue TWAS	
*NQO1*	chr16:69743304-69760463	Multi-tissue TWAS	
*PDXDC2P*	chr16:70,010,201-70,099,851	Multi-tissue TWAS	
*PDPR*	chr16:70147529-70196440	Multi-tissue TWAS	
*DDX19A*	chr16:70380806-70407286	Multi-tissue TWAS	
*COG4*	chr16:70514470-70557457	Multi-tissue TWAS	
***SENP3***	**chr17:7465236-7475287**	Multi-tissue TWAS	**novel**
*UTP6*	chr17:30187923-30228727	Multi-tissue TWAS	*RHOT1-RHBDL3 -* PMID: 34127677
*RHBDL3*	chr17:30592851-30651678	Multi-tissue TWAS	
*RHOT1*	chr17:30469521-30552746	both	
*AC116407.1*	chr17:30462748-30462833	Tissue-specific TWAS	
*CNTNAP1*	chr17:40834549-40852011	Multi-tissue TWAS	near *MIR2117HG -* PMID: 34127677
*ARL4D*	chr17:41476361-41478505	Tissue-specific TWAS	
***GOSR2***	**chr17:45000526-45014188**	Multi-tissue TWAS	**novel**
***DDX5***	**chr17:62494374-62502484**	Multi-tissue TWAS	**novel**
***CEP95***	**chr17:62503095-62534064**	Tissue-specific TWAS	**novel**
***AC007773.1***	**chr19:32868188-32868273**	Multi-tissue TWAS	**novel**
*NECTIN2*	chr19:45349554-45382195	Multi-tissue TWAS	near *EXOC3L2 -* PMID: 34127677
*JAG1*	chr20:10618332-10654694	Multi-tissue TWAS	*JAG1 -* PMID: 34127677
*SLC24A3*	chr20:19193286-19703570	Multi-tissue TWAS	*SLC24A3 -* PMID: 34127677
***EIF2S2***	chr20:32676115-32700085	Multi-tissue TWAS	**novel**
***ASIP***	chr20:32848171-32857148	Multi-tissue TWAS	
*MTMR3*	chr22:30279163-30426857	Multi-tissue TWAS	*HORMAD2 -* PMID: 34127677

Genes in bold are novel (i.e., no prior reported GWAS SNV within 1 Mb).

Interestingly, 12 genes were Bonferroni-significant in only one tissue reference panel; these included 10 genes within previously reported cataract-associated loci: *ARL4D* (thyroid); *CDC42BPA* (heart atrial appendage); *CDKN2A* (brain cortex); *GSTM2* (whole blood); *OCA2* (brain); *PKD2L1* (lung); *SEMA4D* (spleen); *ST6GALNAC4* (blood); *TESMIN* (adipose subcutaneous); and *TPCN2* (brain cerebellar hemisphere); and 2 genes newly identified in the current study: *CEP95* (skin sun exposed lower leg), and *INSRR* (brain nucleus accumbens basal ganglia) **Supplementary Table S2**.

To assess whether common genetic variants underly eQTL and GWAS associations with cataract, we conducted a colocalization analysis for the 202 Bonferroni-significant gene-tissue pairs. We found that 128 (63.4%) of the Bonferroni-significant gene-tissue pairs had a colocalized variant associated with both cataract risk (from GWAS) and predicted gene expression based on our TWAS results (column COLOC.PP4 in **Supplementary Table S2**).

### Conditional analyses provide additional support for cataract TWAS associations

To identify TWAS signals for cataract independent of GWAS genome-wide significant risk variants, we repeated the FUSION analysis with GWAS summary statistics conditioned on the top GWAS SNV in each of the 202 Bonferroni-significant gene-tissue pairs. We found that all gene-tissue pairs reached nominal significance (*P* < 0.05) (**Supplementary Table S2**). Furthermore, we assessed joint TWAS associations in tissue reference panels with more than one Bonferroni-significant gene on the same chromosome region (**Supplementary Table S3**). Of the four pairwise joint models including six unique genes, all the associations were attenuated but retained marginal significance (P<0.05). All six of these genes (*IGHMBP2*, *TPCN2*, *MRPL21*, *MRGPRF-AS1*, *AP000808.1*, and *MRGPRD*) are located within the 11q13.3 chromosome region, which has been previously identified as a GWAS susceptibility locus for cataract (11, 12).

### Sex-specific TWAS analyses revealed 9 genes associated with cataract

Because cataracts are more common in women (4) and genetic susceptibility loci specific to women have been previously identified (11), we evaluated sex-specific TWAS associations. We used sex-specific GWAS summary statistics and tissue reference panels, i.e., GWAS summary statistics from women for TWAS of ovary, uterus, and vagina eQTLs; and GWAS summary statistics from men for TWAS of prostate and testis eQTLs. We found that 22 of the sex-specific tests reached the Bonferroni significance level that we applied to the main analysis (P<1.54x10^-7^), including 3, 5, and 4 genes for ovary, uterus, and vagina, respectively, and 7 and 3 genes for prostate and testis, respectively (**Supplementary Table S4**). Of those 22 sex-specific associations, 9 unique genes were identified, all of these genes (*ITPKB, AC104162.1, AP000808.1, MRPL21, IGHMBP2, CAPRIN2, CLEC18A, LINC01229* and *AC003681.1*) were located nearby previously identified GWAS loci for cataract (11, 12). For instance, while differential gene expression of *MRPL21* at 11q13.3 was associated with cataract in the 5 sex-specific tissue reference panels (i.e., ovary, uterus, vagina, prostate, and testis), differential gene expression of *ITPKB* was associated with cataract in vagina only.

### Importance of gastrointestinal tissues in cataract susceptibility

Across the 54 tissue reference panels, the greatest number of Bonferroni-significant gene-tissue pairs was observed for the GTEx adipose subcutaneous, artery tibial, and thyroid datasets (seven gene-tissue pairs for each dataset), followed by the GTEx artery aorta, heart atrial appendage, esophagus muscularis, and skin sun exposed datasets (six gene-tissue pairs for each dataset) (**Supplementary Table S1**). To identify tissues potentially relevant to cataract, we assigned tissue reference panels to anatomical categories as described above in the Methods (**Supplementary Table S1**). In the tissue-specific TWAS results, gastrointestinal tissues represented one of the highest proportion of the 202 Bonferroni-significant genes (43 genes; 21.3%) (**Supplementary Figure S2**). Interestingly, this gastrointestinal tissue type was the only anatomical category significantly enriched in our results, after accounting for the number of gene-tissue pairs tested per anatomical category (p-value from the hypergeometric test = 0.0055) (**Supplementary Table S5**).

### Multi-tissue TWAS revealed additional novel candidate genes for cataract susceptibility

The multi-tissue TWAS using the omnibus test in FUSION revealed 86 genes for which imputed expression was associated with cataract susceptibility (Bonferroni p-value <0.05/13,328 effective gene tests ≈ 3.75x10^-6^ and minimum tissue-specific p-value < 1 x 10^-5^) (**Supplementary Table S6**). Interestingly, 14 of the 86 multi-tissue associated genes were also associated with cataract in tissue-specific models, including *INSRR* (chr1), *CDC42BPA* (chr1), *CDKN2A* (chr9), *CDKN2B* (chr9), *SEMA4D* (chr9), *ST6GALNAC4* (chr9), *PKD2L1* (chr10), *TESMIN* (chr11), *IGHMBP2* (chr11), *BMP4* (chr14), *OCA2* (chr15), *TBX6* (chr16), *RHOT1* (chr17) and*ARL4D* (chr17). In addition, 18 of the 86 multi-tissue associated genes were located outside of previously described risk loci (**Table 1**). These included: *CROCC* (chr1), *ATP13A2* (chr1), *CAPZB* (chr1), *SH3YL1* (chr2), *EFEMP1* (chr2), *CREB1* (chr2), *ANKH* (chr5), *NIPSNAP2* (chr7), *HERC4* (chr10), *PICALM* (chr11), *COQ6* (chr14), *ESRRB* (chr14), *SENP3* (chr17), *GOSR2* (chr17), *DDX5* (chr17), *AC007773.1* (chr19), *EIF2S2* (chr20), and *ASIP* (chr20).

### Gene expression in the lens tissue

**Figure 3:**
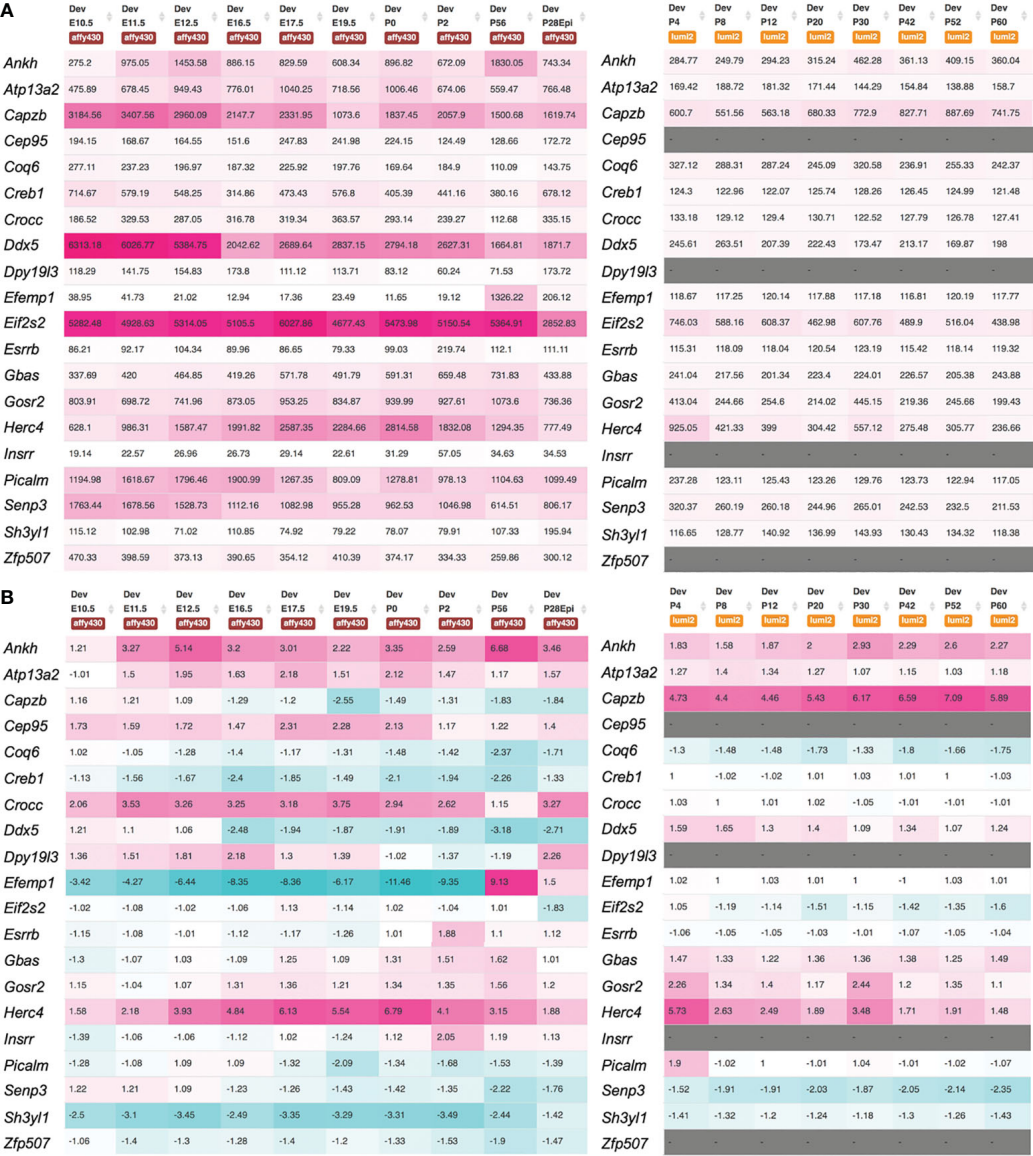
Expression of novel candidate genes for cataract in the mouse lens. Mouse orthologs of the human candidate genes were examined for their lens expression in the iSyTE microarray datasets. **(A)** Analysis of whole lens tissue data on the Affymetrix and Illumina microarray platforms at different embryonic (E) and postnatal (P) stages indicates that majority of the candidates are expressed in the lens. The heat-map denotes the range of expression on either the Affymetrix or Illumina platform, while the number represents the mean fluorescence intensity for individual genes. **(B)** Mouse orthologs of the human candidate genes were examined for their “lens enriched” expression in the iSyTE microarray data. “Enriched expression” in the lens is estimated by analyzing the fold-change enrichment of candidate gene expression in the lens compared to that in whole embryonic body as indicated by the number and heatmap. Please note that WEB in the Affymetrix data represents expression in whole embryonic body in **(A)** and P28Epi in the Affymetrix data represents expression data on isolated lens epithelium in **(A, B)**.

We identified the mouse orthologs for 19 of the 20 novel genes as follows. For *NIPSNAP2*, in the Affy and Illumina microarray data, the gene alias for mouse *Gbas* was used. For AC007773.1, *ZNF507* (mouse ortholog, *Zfp507*) and *DPY19L3* (mouse ortholog, *Dpy19l3*) were considered as candidate genes. We first examined the expression of these genes in the lens tissue across various stages using the iSyTE microarray database (21, 22). While majority of the genes were found to be expressed, several exhibited robust expression (**Figure 3A**). For example, *Atp13a2*, *Capzb*, *Crocc*, *Efemp1*, *Gbas*, *Gosr2*, *Picalm*, *Senp3* and *Zfp507* had high expression in Affymetrix datasets. When examined for “enriched expression” in the lens, several candidates (*e.g.*, *Atp13a2*, *Capzb*, *Cep95*, *Crocc*, *Dpy19l3*, *Efemp1*, *Esrrb*, *Gbas*, *Gosr2*, *Insrr*, *Picalm*, and *Senp3*) were identified (**Figure 3B**). Moreover, RNA-seq data from whole lens tissue confirmed 10 of the mouse orthologs to have expression or enriched expression in the lens (**Supplementary Figures S3, 4**). Further, expression data from isolated lens epithelium and fiber cells at different time-points, spanning embryonic (E14.5 through newborn) through aging stages (3 months through age 2 years) showed that all the novel cataract candidate genes with mouse orthologs exhibit robust expression in the epithelium and/or fiber cells (**Supplementary Figure S5**). Interestingly, this cell-specific data also shows that three candidate genes are specifically enriched in the postnatal epithelium (*e.g.*, *Efemp1*, *Esrrb*, *Insrr*) and a subset of these exhibit progressively high expression with aging in the epithelium (*e.g.*, *Efemp1*, *Insrr*). Finally, all novel candidate genes exhibited differential expression in at least one gene-perturbation mouse models of lens defects/cataract (**Supplementary Figure S6**).

## Discussion

By leveraging data from GWAS and eQTL, we identified 99 genes associated with cataract susceptibility (16 from the tissue-specific analysis alone, 69 from the multi-tissue analysis alone, and 14 from both analyses). Of these 99 genes, 20 were novel to the extent they did not overlap known cataract risk loci from GWAS *(*2 of these*, INSRR and CEP95*, were from tissue-specific models, and 18 from the multi-tissue analysis). Mouse orthologs of the vast majority of the human candidate genes were found to be robustly expressed in the lens. We also highlighted the contribution of the 11q13.3 genomic region in cataract susceptibility. Our results implicated a role for gastrointestinal tissues and confirmed the importance of the lens in cataract etiology.

Our tissue-specific TWAS analysis identified *INSRR* (1q23.1) and *CEP95* (17q23.3) as novel cataract-associated genes. *INSRR* encodes the insulin receptor related receptor which is involved in the transmembrane receptor protein tyrosine kinase activity, actin cytoskeleton reorganization, and the protein autophosphorylation, and has an important role in the alkaline pH-dependent activation mechanism (43). *CEP95* encodes the centrosomal protein 95 and belongs to the family of proteins containing coiled-coil domains (CCDCs), which are involved in several functions in cell growth and development, such as regulation of gene expression (44). To date, no mutations in either *INSRR* or *CEP95* have been linked to eye diseases, and further studies are needed to confirm the role of these genes in cataract etiology and determine their precise role in cataract susceptibility.

Our multi-tissue TWAS analysis identified *ATP13A2* (1p36.13), *CAPZB* (1p36.13), *EFEMP1* (2p16.1), and *SENP3* (17p13.1) associated with cataract, all were not previously reported as significant in GWA studies of cataract. *ATP13A2* encodes a member of the P5 subfamily of ATPases which transports inorganic cations as well as other substrates. The *ATP13A2* locus has been previously reported to be associated with age-related cataract in a GWAS conducted in a Chinese cohort (45); however, this association did not reach a genome-wide level of significance (lead SNV rs2871776, *P*=4.18x10^-5^), possibly due to limited sample size (total of 191 cataract cases and 208 controls) (45). *CAPZB* encodes the beta subunit of the barbed-end actin binding protein, which belongs to the F-actin capping protein family. Interestingly, *CAPZB* is located within the 1p36 chromosome region that was previously linked to congenital cataract in three genetic linkage studies (46–48). However, no segregating mutations that contribute to congenital cataract were identified in this *CAPZB* gene in a six-generation Australian family displaying linkage to chromosome 1p36 (49). *EFEMP1* encodes a member of the fibulin family of extracellular matrix glycoproteins, and mutations in this gene have been shown to cause Doyne honeycomb retinal dystrophy and familial juvenile-onset open-angle glaucoma (50, 51). Recently, *EFEMP1* has been demonstrated to be a potential biomarker for choroidal neovascularization in age-related macular degeneration, and for choroidal thickness change in myopia (52, 53). Interestingly, a transcriptome analysis of neural progenitor cells derived from patients with Lowe syndrome, a multisystem disorder characterized notably by anomalies affecting the eye, including congenital cataracts, identified *EFEMP1* as a candidate gene (54). Future studies will clarify how *EFEMP1* contributes to cataract susceptibility. Our study also identified *SENP3* as a cataract-associated gene. *SENP3* encodes the SUMO specific peptidase 3 which is a de-sumoylation enzyme (SENP) that plays an important role in regulating eye development and is highly expressed in vertebrate ocular cell lines, including human, mouse, and rabbit lens epithelial cells lines (55, 56). Previous works have demonstrated that sumoylation function plays indispensable roles during lens differentiation (57, 58). Recently, glucose oxidase and UVA irradiation seem to affect the expression patterns of the *SENP*s, including *SENP3*, in the *in vitro* cataract models, providing evidence to link sumoylation function to stress-induced cataractogenesis (59). Moreover, the changing patterns in some *SENP*s levels seem to act as molecular markers for both senile and complicated cataracts (60). Additional studies investigating the sumoylation functions and the related mechanisms in cataract development and progression will help to understand the role of *SENP3* in cataract susceptibility.

Among the 9 unique genes identified in the tissue-sex specific analyses of cataract, we identified *ITPKB* (1q42.12), which was differentially expressed in the vagina. *ITPKB* encodes the inositol-trisphosphate 3-kinase B that plays an important role in the regulation of the levels of a large number of inositol polyphosphates (61). A *de novo* 5.8-Mb deletion encompassing the chromosome 1q42.12q42.2 region (and *ITPKB*, among other genes) was reported in a 4-year-old child who presented hypoplastic corpus callosum and bilateral cataracts, in addition to other clinical features such as epilepsy (62). Future investigations may determine the implication of *ITPKB* in cataract etiology.

Our study should be interpreted within the context of its limitations. Although GTEx data for the 49 tissues represent the most comprehensive eQTL dataset of human tissues, it does not include ocular tissues and consequently we may have failed to identify the real causal genes in the unsampled ocular tissue. However, we have confirmed using the iSyTE database that novel cataract genes identified in the current study are robustly expressed in lens tissue, which is a cataract relevant eye tissue. Moreover, although cataract is primarily a lens disorder, it has been demonstrated that most complex diseases, including vision disorders such as age-related macular degeneration, might manifest in several tissues across the body (63). Despite the great success in prioritizing gene-trait associations in complex diseases and traits, TWAS may present multiple hits per locus, owing to co-regulation, which remain problematic (14, 19). Thus, future models could consider more complex genetic architecture containing different regulatory effects, and our TWAS results could benefit from subsequent functional assays to indicate the potential targets underlying the identified associations, notably at 11q13.3. Despite these limitations, our TWAS study is based on results from a large GWAS meta-analysis on almost 60,000 cataract cases, enabling the prioritization and the discovery of potential causal genes for cataract. Finally, in the current study, we performed a multi-tissue TWAS analysis which enables increased statistical precision compared to single-tissue approaches (17, 64, 65).

Our study also highlighted the important contribution of gastrointestinal tissues in cataract susceptibility consistent with previous work showing associations between cataracts and gastrointestinal disorders (66, 67). For instance, patients diagnosed with early-onset cataracts have been shown to be at increased risk of peptic ulcer (66). Furthermore, rare syndromic disorders for which patients present early-onset congenital cataracts can present gastrointestinal disorders as additional features (68–71). For instance, patients with Lowe Syndrome (oculocerebrorenal syndrome) can present both dense congenital cataracts and gastroesophageal reflux (68). Similarly, patients with inherited spastic paraplegia can present with bilateral cataracts and gastroesophageal reflux with persistent vomiting (69). A splice site mutation in *CYP27A1* has been reported to lead to cerebrotendinous xanthomatosis which can be characterized by pulverulent cataracts and gastrointestinal problems such as diarrhea (71). Recently, pathogenic variants in the *WFS1/RP1/NOD2* genes have been shown to cause congenital cataract, retinitis pigmentosa, and Crohn’s disease in a five generation British family (70). A comprehensive evaluation of systemic disorders associated with age-related cataract – as previously done for dry eye disease (72)- would help to identify which gastrointestinal disorders are risk factors for cataract. Altogether, expression of genes associated with cataract seems not to be restricted to lens tissue, as could be expected for this lens disorder, and the processes underlying cataract pathology seem to be systemic as observed for other vision disorders, such as age-related macular degeneration and exfoliation syndrome (37, 73).

In conclusion, we identified 99 genes associated with cataract susceptibility, of which 20 did not overlap with known cataract risk loci. Our results provide evidence of the utility of imputation-based TWAS approaches to characterize known GWAS risk loci and identify novel candidate genes that may increase our understanding of cataract etiology.

## Data availability statement

The original contributions presented in the study are publicly available. FUSION models trained on the GTEx version 8 data are available here: http://gusevlab.org/projects/fusion/. Gene expression and eQTL data are freely available at https://gtexportal.org/home/datasets. Expression or lens-enriched expression heat-map for candidate genes can be accessed through the iSyTE web-tool (https://research.bioinformatics.udel.edu/iSyTE). The RNA-seq data can be found here: Gene Expression Omnibus (GEO; GSE113887, GSE166619, GSE119596). The microarray data can be found here: Gene Expression Omnibus (GEO; GSE100136, GSE32334, GSE65500, GSE47694, GSE16533, GSE31643, GSE9711, GSE13402, GSE25775, GSE25776, GSE22322, GSE22362, GSE9711). To protect individual’s privacy, complete GERA data are available upon approved applications to the KP Research Bank Portal (https://researchbank.kaiserpermanente.org/). A subset of the GERA cohort consented for public use can be found at NIH/dbGaP: phs000674.v3.p3.

## Ethics statement

The studies involving humans were approved by The Institutional Review Board of the Kaiser Permanente Northern California. The studies were conducted in accordance with the local legislation and institutional requirements. The participants provided their written informed consent to participate in this study. The animal study was approved by The University of Delaware Institutional Animal Care and Use Committee (IACUC). The study was conducted in accordance with the local legislation and institutional requirements.

## Author contributions

HC: Conceptualization, Funding acquisition, Resources, Supervision, Visualization, Writing – original draft, Writing – review & editing. MD: Formal analysis, Visualization, Writing – review & editing. VH: Formal analysis, Visualization, Writing – review & editing. SS: Formal analysis, Visualization, Writing – review & editing. TM: Conceptualization, Methodology, Writing – review & editing. TH: Methodology, Resources, Writing – review & editing. PS: Conceptualization, Writing – review & editing. SL: Conceptualization, Funding acquisition, Resources, Supervision, Visualization, Writing – original draft, Writing – review & editing.
