# Supplementary material for: Multi-tissue transcriptome-wide association study identifies novel candidate susceptibility genes for cataract
Source: Front Ophthalmol (Lausanne). 2024 Apr 16;4:1362350. doi: 10.3389/fopht.2024.1362350 (PMC11182099; doi:10.3389/fopht.2024.1362350)
Supplement: Supplementary file 2 [file DataSheet_2.pdf]

Choquet et al. "Multi-tissue transcriptome-wide association study identifies novel candidate susceptibility genes for cataract"

Supplementary Figures

Supplementary Figure S1. Flowchart of the study design.

Supplementary Figure S2. Bonferroni-significant TWAS cataract genes by anatomical category.

Supplementary Figure S3. Expression and enriched-expression of candidate cataract genes in whole lens RNA-seq data.

Supplementary Figure S4. Expression heatmap of novel cataract candidate genes in mouse lens isolated epithelium and fibers RNA-seq data.

Supplementary Figure S5. Expression of novel cataract candidate genes in mouse lens isolated epithelium and fibers.

Supplementary Figure S6. Expression of novel cataract candidate genes is altered in various gene perturbation mouse models with lens defects.

Supplementary Figure S1. Flowchart of the study design. This figure summarizes the different data sources and main analyses/results of the current TWAS study.

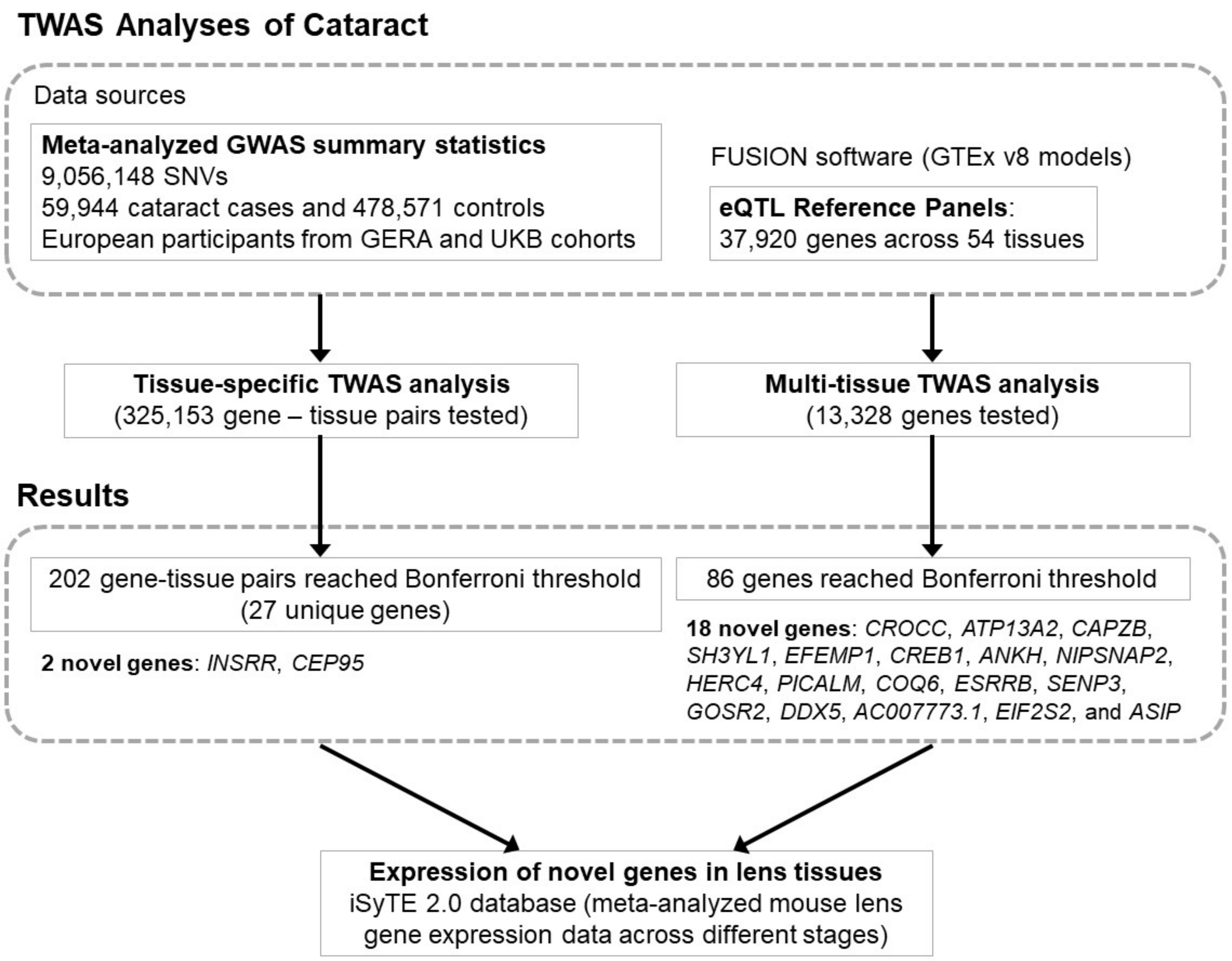

Supplementary Figure S2. Bonferroni-significant TWAS cataract genes by anatomical category. Gastrointestinal tissue type was the only anatomical category significantly enriched in our results, after correcting for the number of tissue donors and imputable genes for each reference panel.

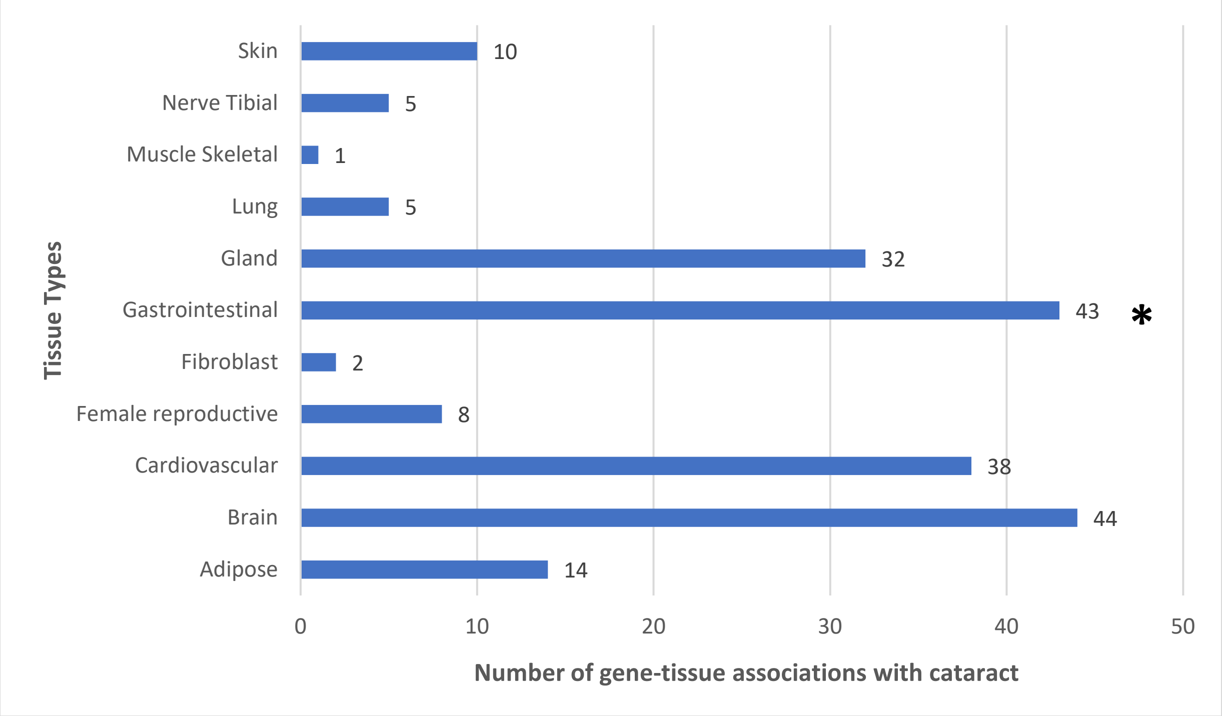

Figure Legend: In the tissue-specific TWAS, 202 gene-tissue associations with cataract were Bonferroni-significant. To compare these TWAS associations by anatomical category, eQTL reference tissues were grouped where appropriate (see **Supplementary Table S1**). The asterisk indicates gastrointestinal tissue as the only category with a hypergeometric test p-value less than 0.05 accounting for the number of gene-tissue pairs tested per category.

Supplementary Figure S3. Expression and enriched-expression of candidate cataract genes in whole lens RNA-seq data. Visualization of RNA-seq lens expression and enrichment of mouse orthologs of human candidate genes using UCSC browser iSyTE custom tracks. Analysis of publicly available RNA-seq datasets informing on gene expression at different stages, namely, embryonic (E) day E10.5, E12.5, E14.5, and E16.5 demonstrates expression or enriched expression of the mouse orthologs of the human candidate genes in whole lens. To access the RNA-seq-based lens expression and lens enrichment data, UCSC custom tracks on the mouse genome GRCm38/mm10 assembly were examined using iSyTE 2.0 at https://research.bioinformatics.udel.edu/iSyTE/. Four tracks each for visual representation of lens gene expression and enriched expression for the different mouse embryonic stages for individual candidate genes are given. The heat-map denotes the range of expression or enriched expression. A color key is provided on iSyTE 2.0 to estimate lens enrichment (based on fold-change gene expression in the lens compared to WB) and expression (based on counts for expression). Note: Ankh is denoted as Ank.

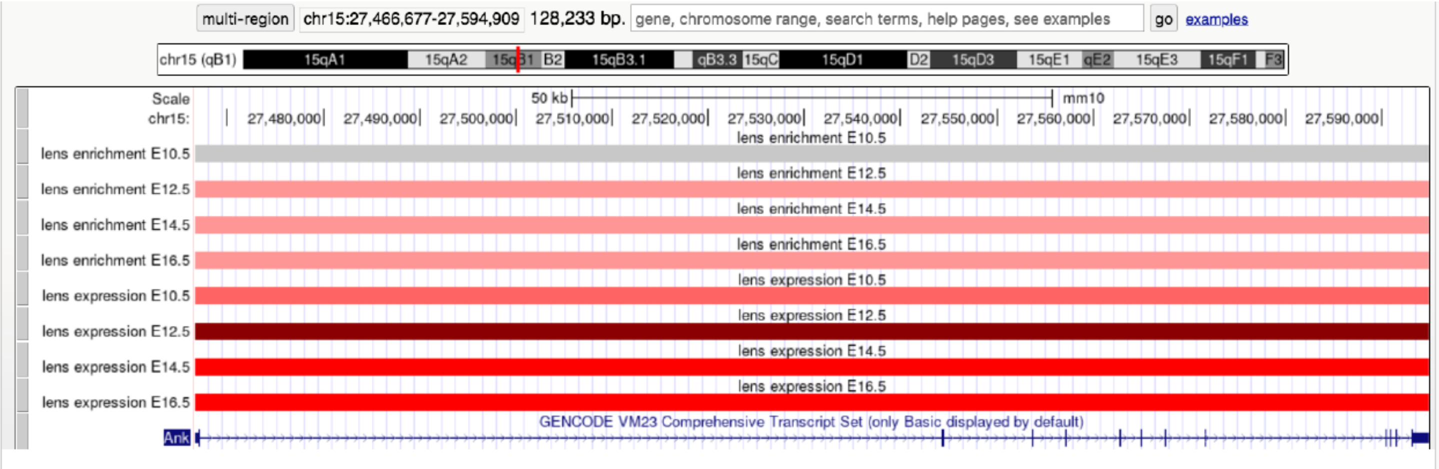

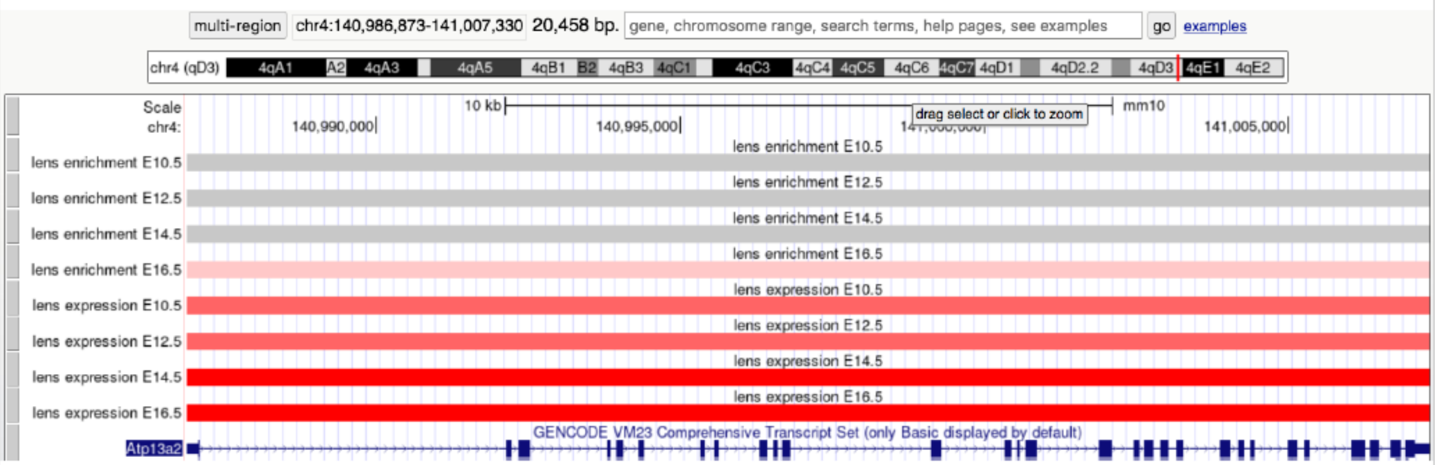
multi-region
chr4:140,986,873-141,007,330
20,458 bp.
gene, chromosome range, search terms, help pages, see examples
go
examples
chr4 (qD3)
Scale
chr4:
10 kb
mm10
drag select or click to zoom
140,990,000
140,995,000
141,005,000
lens enrichment E10.5
lens enrichment E12.5
lens enrichment E14.5
lens enrichment E16.5
lens expression E10.5
lens expression E12.5
lens expression E14.5
lens expression E16.5
GENCODE VM23 Comprehensive Transcript Set (only Basic displayed by default)
Atp13a2

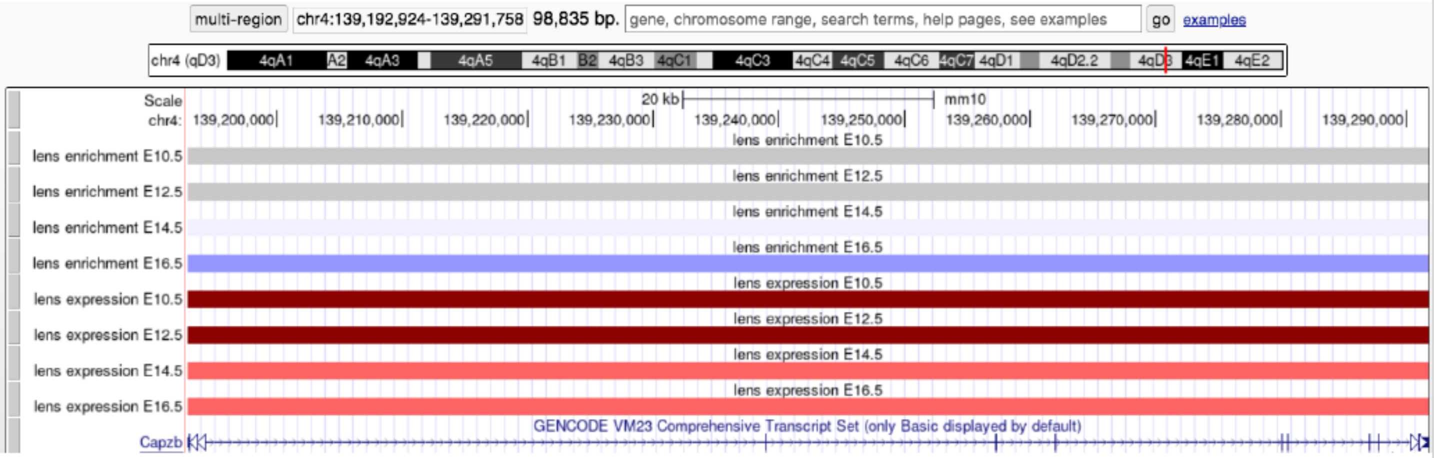
multi-region
chr4:139,192,924-139,291,758
98,835 bp.
gene, chromosome range, search terms, help pages, see examples
go
examples
chr4 (qD3)
Scale
chr4:
20 kb
mm10
139,200,000
139,210,000
139,220,000
139,230,000
139,240,000
139,250,000
139,260,000
139,270,000
139,280,000
139,290,000
lens enrichment E10.5
lens enrichment E12.5
lens enrichment E14.5
lens enrichment E16.5
lens expression E10.5
lens expression E12.5
lens expression E14.5
lens expression E16.5
GENCODE VM23 Comprehensive Transcript Set (only Basic displayed by default)
Capzb

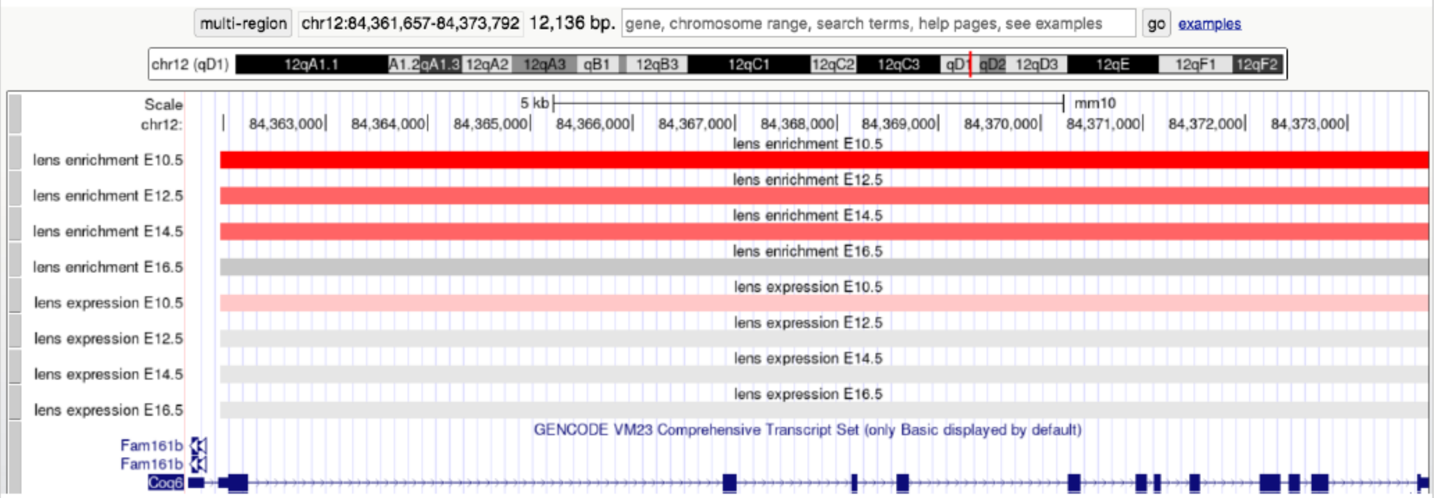
multi-region
chr12:84,361,657-84,373,792
12,136 bp.
gene, chromosome range, search terms, help pages, see examples
go
examples
chr12 (qD1)
Scale
chr12:
5 kb
mm10
84,363,000
84,364,000
84,365,000
84,366,000
84,367,000
84,368,000
84,369,000
84,370,000
84,371,000
84,372,000
84,373,000
lens enrichment E10.5
lens enrichment E12.5
lens enrichment E14.5
lens enrichment E16.5
lens expression E10.5
lens expression E12.5
lens expression E14.5
lens expression E16.5
GENCODE VM23 Comprehensive Transcript Set (only Basic displayed by default)
Fam161b
Fam161b
Coq6

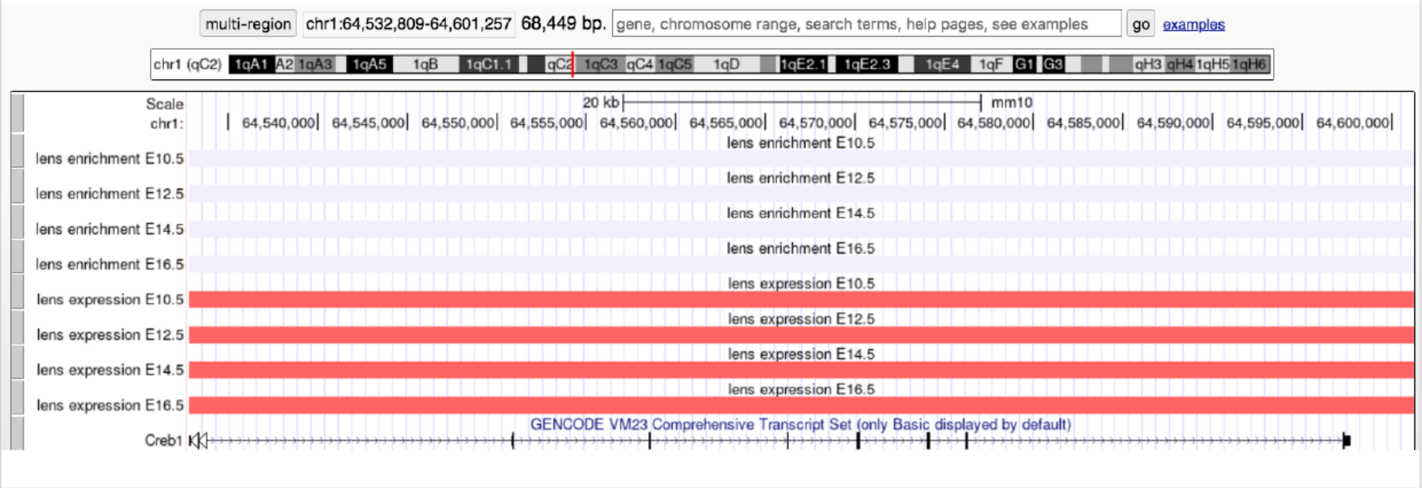

multi-region
chr1:64,532,809-64,601,257
68,449 bp.
gene, chromosome range, search terms, help pages, see examples
go
examples
Scale
chr1:
20 kb
mm10
lens enrichment E10.5
lens enrichment E12.5
lens enrichment E14.5
lens enrichment E16.5
lens expression E10.5
lens expression E12.5
lens expression E14.5
lens expression E16.5
GENCODE VM23 Comprehensive Transcript Set (only Basic displayed by default)
Creb1

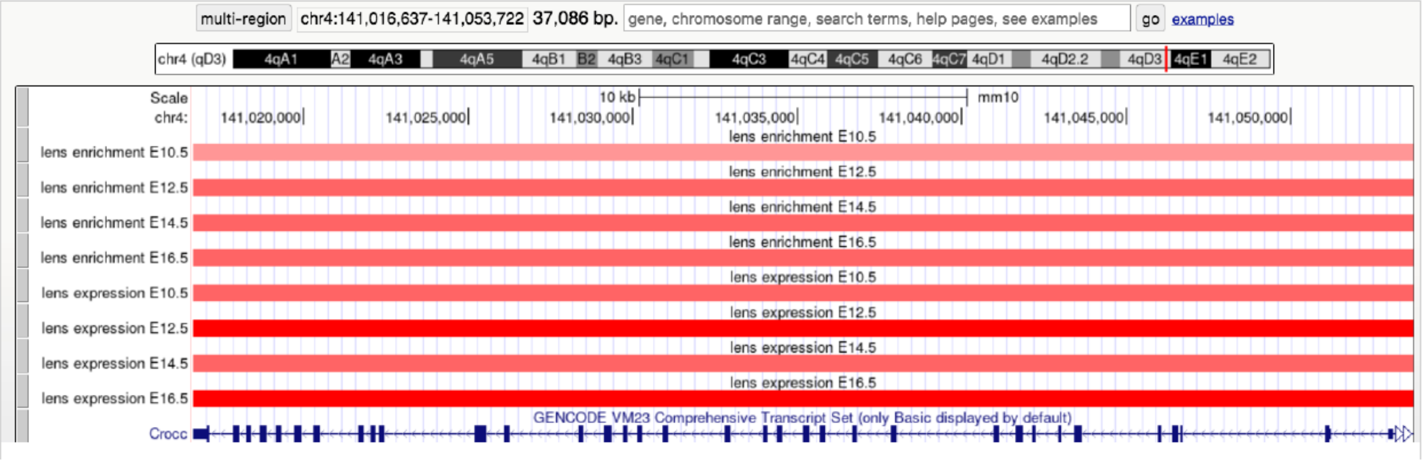

multi-region
chr4:141,016,637-141,053,722
37,086 bp.
gene, chromosome range, search terms, help pages, see examples
go
examples
Scale
chr4:
10 kb
mm10
lens enrichment E10.5
lens enrichment E12.5
lens enrichment E14.5
lens enrichment E16.5
lens expression E10.5
lens expression E12.5
lens expression E14.5
lens expression E16.5
GENCODE VM23 Comprehensive Transcript Set (only Basic displayed by default)
Crocc

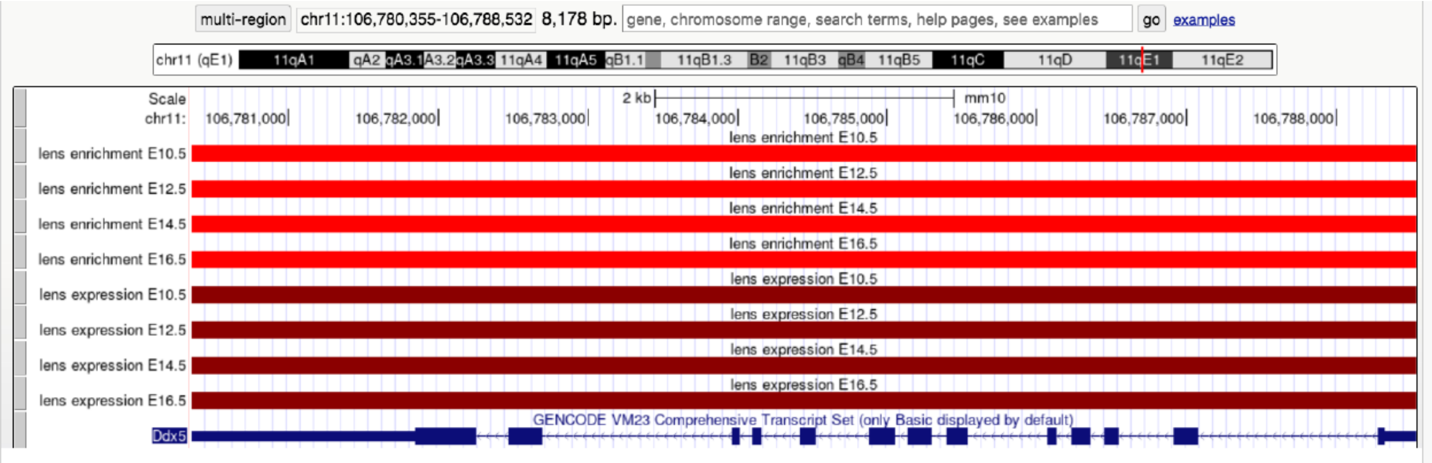

multi-region
chr11:106,780,355-106,788,532
8,178 bp.
gene, chromosome range, search terms, help pages, see examples
go
examples
Scale
chr11:
2 kb
mm10
lens enrichment E10.5
lens enrichment E12.5
lens enrichment E14.5
lens enrichment E16.5
lens expression E10.5
lens expression E12.5
lens expression E14.5
lens expression E16.5
GENCODE VM23 Comprehensive Transcript Set (only Basic displayed by default)
Ddx5

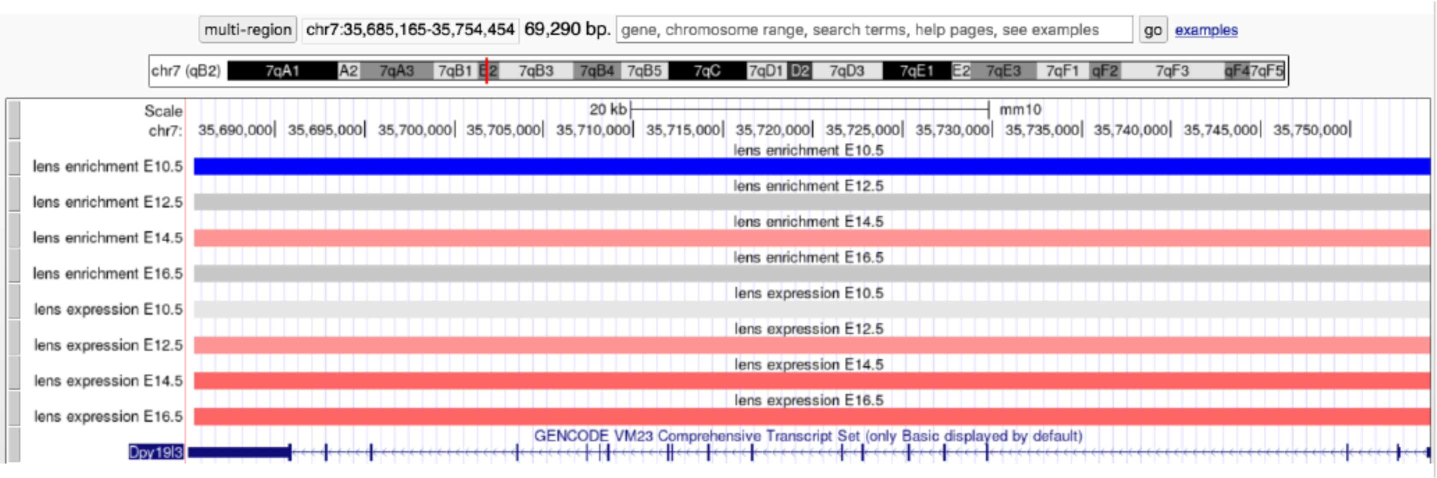
multi-region
chr7:35,685,165-35,754,454
69,290 bp.
gene, chromosome range, search terms, help pages, see examples
go
examples
chr7 (qB2)
Scale
20 kb
mm10
chr7:
35,690,000 35,695,000 35,700,000 35,705,000 35,710,000 35,715,000 35,720,000 35,725,000 35,730,000 35,735,000 35,740,000 35,745,000 35,750,000
lens enrichment E10.5
lens enrichment E12.5
lens enrichment E14.5
lens enrichment E16.5
lens expression E10.5
lens expression E12.5
lens expression E14.5
lens expression E16.5
GENCODE VM23 Comprehensive Transcript Set (only Basic displayed by default)
Dpy19l3

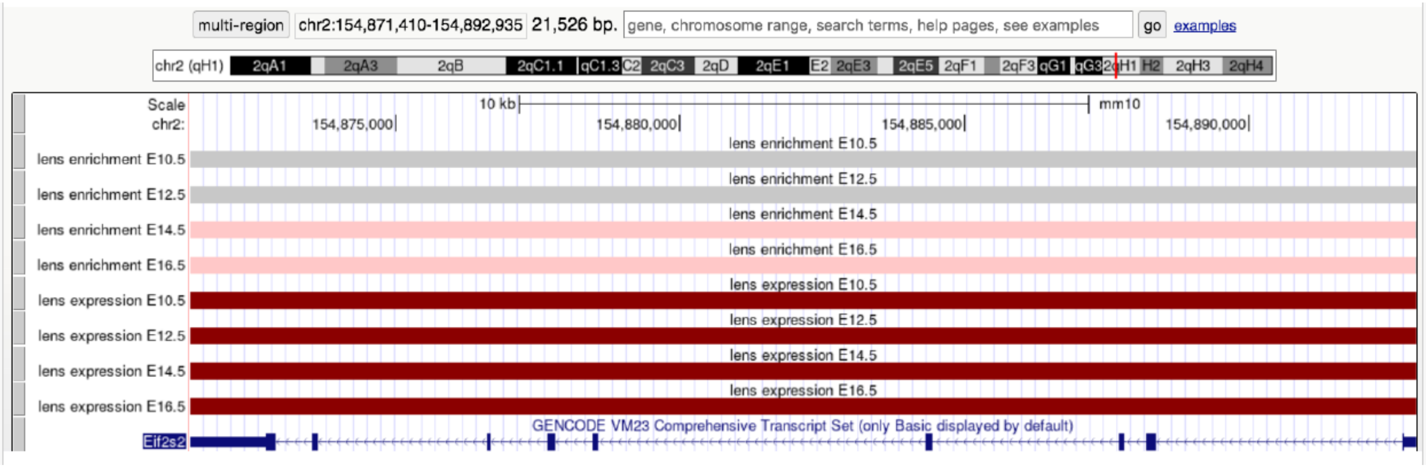
multi-region
chr2:154,871,410-154,892,935
21,526 bp.
gene, chromosome range, search terms, help pages, see examples
go
examples
chr2 (qH1)
Scale
10 kb
mm10
chr2:
154,875,000 154,880,000 154,885,000 154,890,000
lens enrichment E10.5
lens enrichment E12.5
lens enrichment E14.5
lens enrichment E16.5
lens expression E10.5
lens expression E12.5
lens expression E14.5
lens expression E16.5
GENCODE VM23 Comprehensive Transcript Set (only Basic displayed by default)
Eif2s2

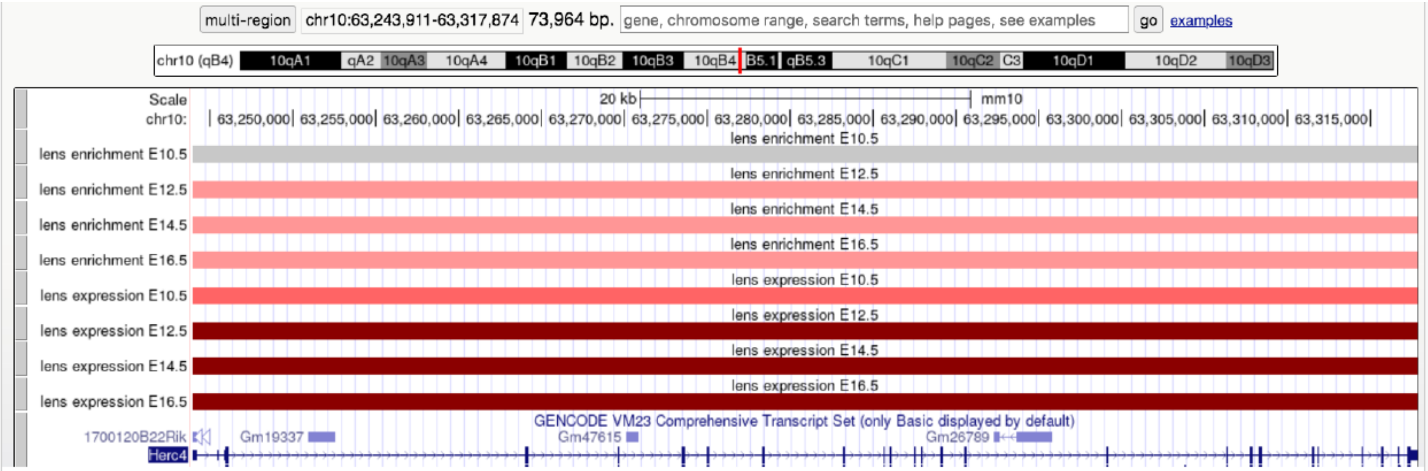
multi-region
chr10:63,243,911-63,317,874
73,964 bp.
gene, chromosome range, search terms, help pages, see examples
go
examples
chr10 (qB4)
Scale
20 kb
mm10
chr10:
63,250,000 63,255,000 63,260,000 63,265,000 63,270,000 63,275,000 63,280,000 63,285,000 63,290,000 63,295,000 63,300,000 63,305,000 63,310,000 63,315,000
lens enrichment E10.5
lens enrichment E12.5
lens enrichment E14.5
lens enrichment E16.5
lens expression E10.5
lens expression E12.5
lens expression E14.5
lens expression E16.5
GENCODE VM23 Comprehensive Transcript Set (only Basic displayed by default)
1700120B22Rik
Gm19337
Gm47615
Gm26789
Herc4

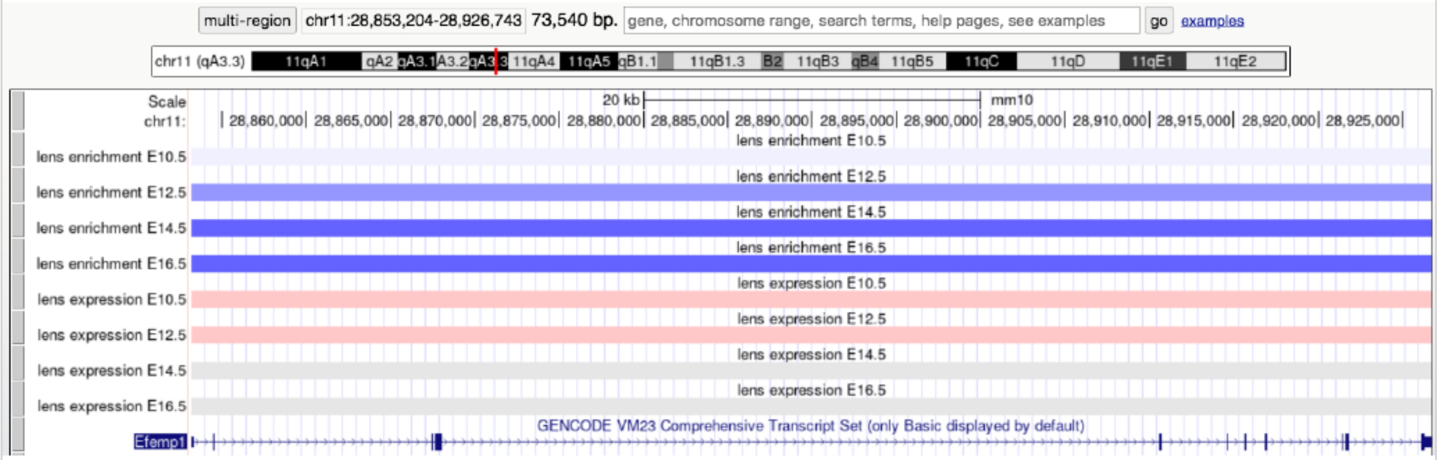
multi-region
chr11:28,853,204-28,926,743
73,540 bp.
gene, chromosome range, search terms, help pages, see examples
go
examples
chr11 (qA3.3)
11qA1
qA2
qA3.1
A3.2
11qA4
11qA5
qB1.1
11qB1.3
B2
11qB3
qB4
11qB5
11qC
11qD
11qE1
11qE2
Scale
chr11:
20 kb
mm10
28,860,000
28,865,000
28,870,000
28,875,000
28,880,000
28,885,000
28,890,000
28,895,000
28,900,000
28,905,000
28,910,000
28,915,000
28,920,000
28,925,000
lens enrichment E10.5
lens enrichment E12.5
lens enrichment E14.5
lens enrichment E16.5
lens expression E10.5
lens expression E12.5
lens expression E14.5
lens expression E16.5
GENCODE VM23 Comprehensive Transcript Set (only Basic displayed by default)
Efemp1

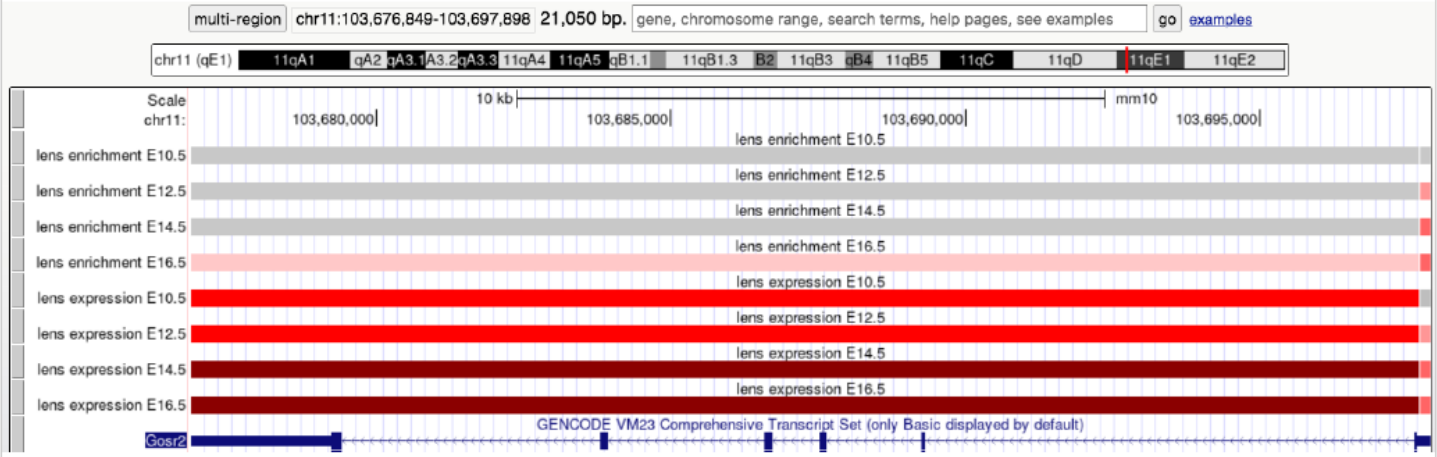
multi-region
chr11:103,676,849-103,697,898
21,050 bp.
gene, chromosome range, search terms, help pages, see examples
go
examples
chr11 (qE1)
11qA1
qA2
qA3.1
A3.2
qA3.3
11qA4
11qA5
qB1.1
11qB1.3
B2
11qB3
qB4
11qB5
11qC
11qD
11qE1
11qE2
Scale
chr11:
10 kb
mm10
103,680,000
103,685,000
103,690,000
103,695,000
lens enrichment E10.5
lens enrichment E12.5
lens enrichment E14.5
lens enrichment E16.5
lens expression E10.5
lens expression E12.5
lens expression E14.5
lens expression E16.5
GENCODE VM23 Comprehensive Transcript Set (only Basic displayed by default)
Gosr2

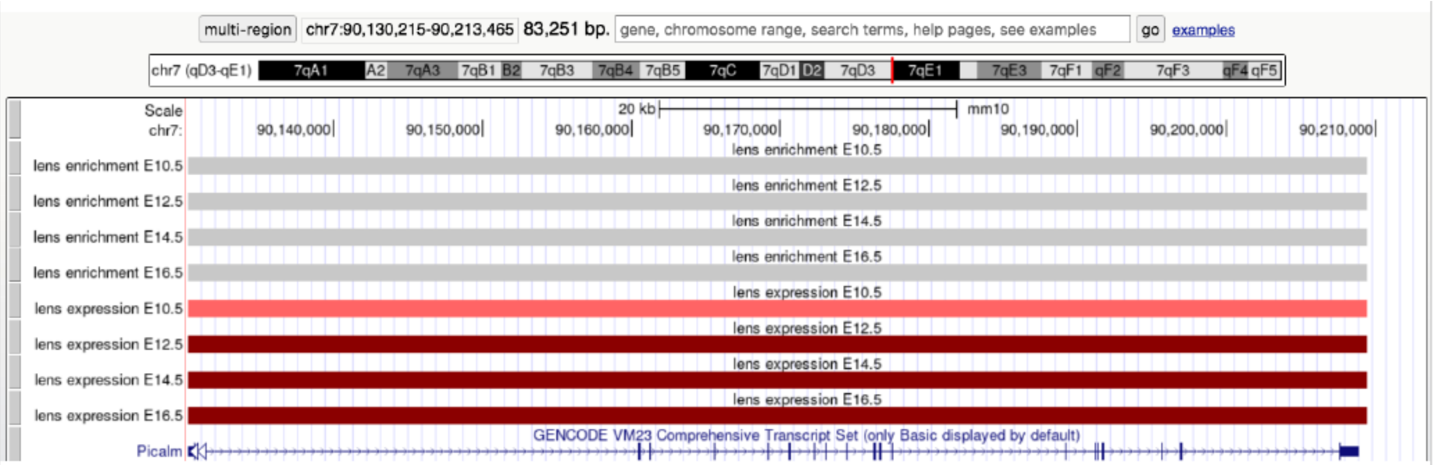
multi-region
chr7:90,130,215-90,213,465
83,251 bp.
gene, chromosome range, search terms, help pages, see examples
go
examples
chr7 (qD3-qE1)
Scale
chr7:
20 kb
mm10
90,140,000
90,150,000
90,160,000
90,170,000
90,180,000
90,190,000
90,200,000
90,210,000
lens enrichment E10.5
lens enrichment E12.5
lens enrichment E14.5
lens enrichment E16.5
lens expression E10.5
lens expression E12.5
lens expression E14.5
lens expression E16.5
GENCODE VM23 Comprehensive Transcript Set (only Basic displayed by default)
Picalm

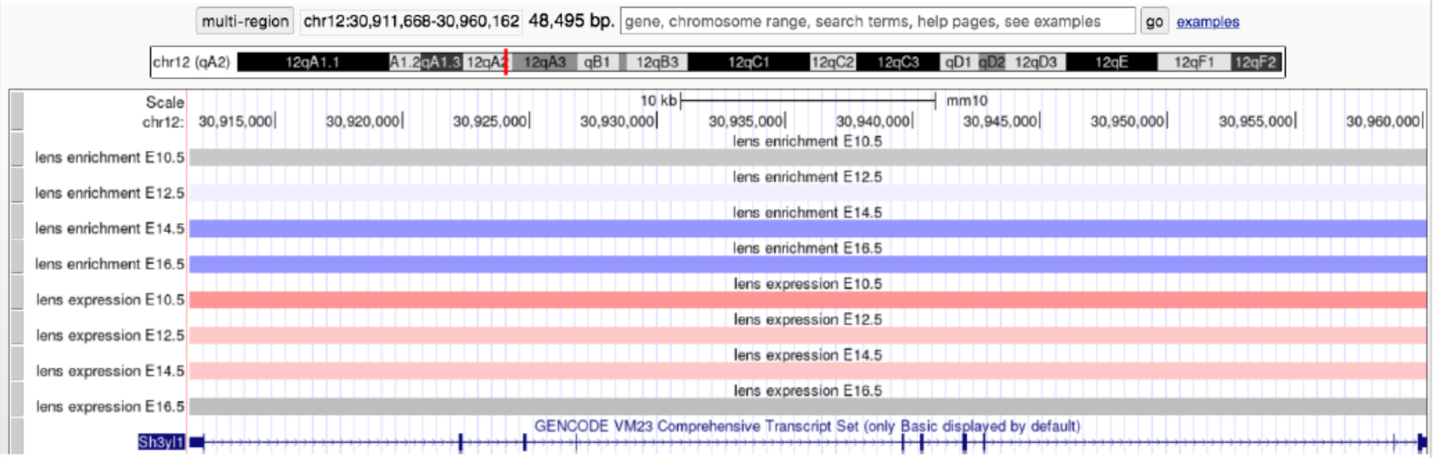
multi-region
chr12:30,911,668-30,960,162
48,495 bp.
gene, chromosome range, search terms, help pages, see examples
go
examples
chr12 (qA2)
Scale
chr12:
10 kb
mm10
30,915,000
30,920,000
30,925,000
30,930,000
30,935,000
30,940,000
30,945,000
30,950,000
30,955,000
30,960,000
lens enrichment E10.5
lens enrichment E12.5
lens enrichment E14.5
lens enrichment E16.5
lens expression E10.5
lens expression E12.5
lens expression E14.5
lens expression E16.5
GENCODE VM23 Comprehensive Transcript Set (only Basic displayed by default)
Sh3yl1

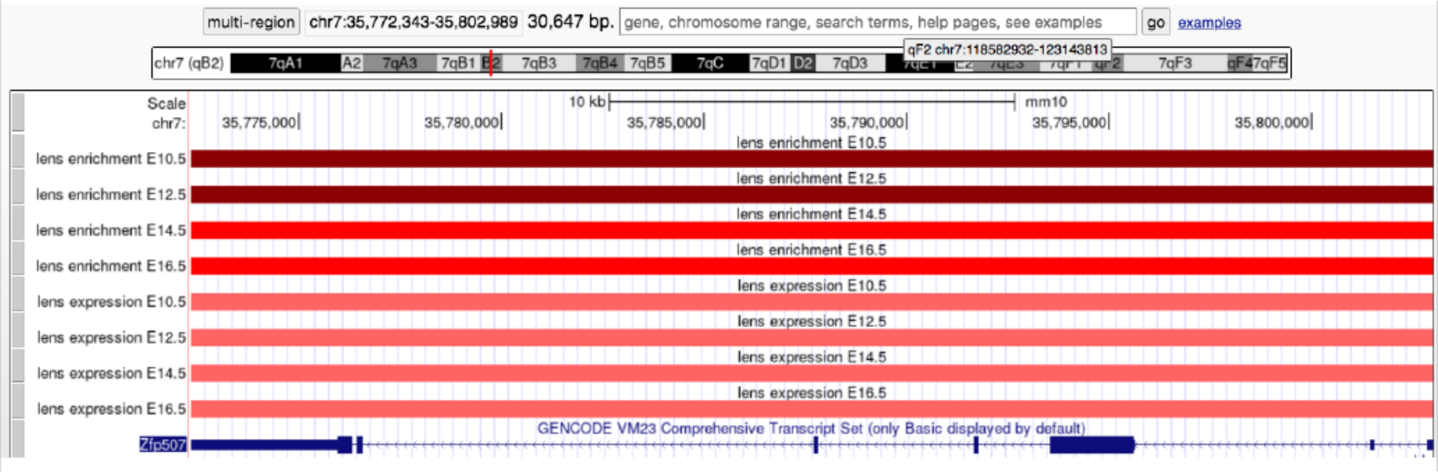
multi-region
chr7:35,772,343-35,802,989
30,647 bp.
gene, chromosome range, search terms, help pages, see examples
go
examples
chr7 (qB2)
qF2 chr7:118582932-123143813
Scale
chr7:
10 kb
mm10
35,775,000
35,780,000
35,785,000
35,790,000
35,795,000
35,800,000
lens enrichment E10.5
lens enrichment E12.5
lens enrichment E14.5
lens enrichment E16.5
lens expression E10.5
lens expression E12.5
lens expression E14.5
lens expression E16.5
GENCODE VM23 Comprehensive Transcript Set (only Basic displayed by default)
Zfp507

Supplementary Figure S4. Expression heatmap of novel cataract candidate genes in mouse lens isolated epithelium and fibers RNA-seq data. Heatmap showing expression of mouse orthologs of the human candidate genes in mouse lens isolated epithelium or fiber cells. Analysis of publicly available RNA-seq datasets informing on gene expression at different stages, namely, embryonic (E) day E14.5, E16.5, E18.5, postnatal (P) day, P0.5 (newborn), age 3 months and age 2 years demonstrates expression of the candidate genes in epithelial and fiber cells. The heat-map denotes the range of expression in either the isolated epithelium or isolated fiber cells, while the number represents the reads per kilobase per million mapped reads (RPKM) values for individual genes.

	EPI E14.5	EPI E16.5	EPI E18.5	EPI P0.5	EPI 3 MO	EPI 2 YR	FC E14.5	FC E16.5	FC E18.5	FC P0.5	FC 3 MO	FC 2YR
Ankh	8504.9	7947.7	5108.8	5862.1	13703.3	15402.3	21980.6	18172.3	16249.2	15342.3	40257.7	21219.7
Atp13a2	10877.3	12919.8	11839.9	11237.2	8151.9	6670.0	27584.4	31751.2	30616.6	31576.3	5896.3	5388.8
Capzb	33881.5	31923.6	31656.3	28859.4	26349.2	27852.2	44926.1	47601.6	53994.5	52881.3	37380.6	41019.5
Cep95	6892.8	6475.2	5595.2	5587.8	2844.2	2815.8	8882.9	8655.6	9057.7	8511.7	3529.9	3594.8
Coq6	3611.9	2904.6	2647.2	2968.5	1452.2	1156.4	3769.3	3721.5	3892.0	4057.8	1181.7	2155.8
Creb1	8346.8	8392.1	8032.0	7355.2	3850.1	3733.3	5845.8	5526.5	5023.8	4415.2	3833.4	3799.1
Crocc	12386.8	11983.5	11670.1	10260.1	20391.3	14467.5	10115.6	9835.1	9148.8	7081.2	8695.6	10668.7
Ddx5	102242.9	113931.9	105824.2	113304.6	104878.1	96785.0	118130.8	131225.7	135732.9	136656.3	65586.7	77078.2
Dpy19l3	5160.5	5878.4	5076.1	4823.4	2951.3	3262.6	4298.1	3811.0	3429.4	2595.6	500.9	562.5
Efemp1	NA	780.7	1727.0	970.3	22486.7	41715.9	NA	15.6	36.9	30.8	656.0	828.6
Eif2s2	18614.9	18440.6	14361.9	13667.4	10402.1	9484.9	34778.5	40440.5	41293.3	40249.8	38542.0	31970.6
Esrrb	NA	NA	NA	NA	3072.7	1581.8	NA	NA	NA	NA	497.3	306.7
Gbas	13887.2	14254.4	14764.9	16176.2	11553.4	12490.8	22755.5	21748.4	21596.1	26211.5	26768.2	22206.8
Gosr2	17382.5	20308.9	19011.1	19500.4	16354.1	18145.3	26188.0	30160.4	32354.8	32474.8	18286.4	20503.0
Herc4	17393.1	20373.6	13091.0	15796.6	11213.5	8449.6	90462.8	97854.5	75628.1	90591.4	28918.6	30254.5
Insrr	NA	NA	NA	NA	433.8	985.4	NA	NA	NA	NA	18.1	0.1
Picalm	12668.7	12104.3	10535.3	10302.7	5834.7	5461.9	16298.1	16953.0	16278.6	15061.4	7234.3	6426.1
Senp3	15884.0	15170.3	13121.7	12717.2	10919.8	11762.4	16549.8	15049.2	13468.8	13094.5	9640.8	11787.8
Sh3yl1	681.3	961.0	1334.0	867.0	591.1	655.6	497.4	392.6	501.9	415.8	350.9	801.0
Zfp507	8260.3	8253.8	7177.8	7510.4	4344.9	3915.0	7993.5	8096.4	8517.2	9031.4	2444.1	3995.1

Supplementary Figure S5. Expression of novel cataract candidate genes in mouse lens isolated epithelium and fibers. Mouse orthologs of the human candidate genes were examined for their expression in mouse lens isolated epithelium or fiber cells. Analysis of publicly available RNA-seq datasets informing on gene expression at different stages, namely, embryonic (E) day E14.5, E16.5, E18.5, postnatal (P) day, P0.5 (newborn), age 3 months and age 2 years demonstrates that all the candidates examined were robustly expressed in either epithelial or fiber cells or both. The Y-axis represents reads per kilobase per million mapped reads (RPKM) values for specific genes. Interestingly, this data shows that some genes are preferentially enriched in epithelium and where their expression increases with age (*e.g.*, *Efemp1*, *Insrr*).

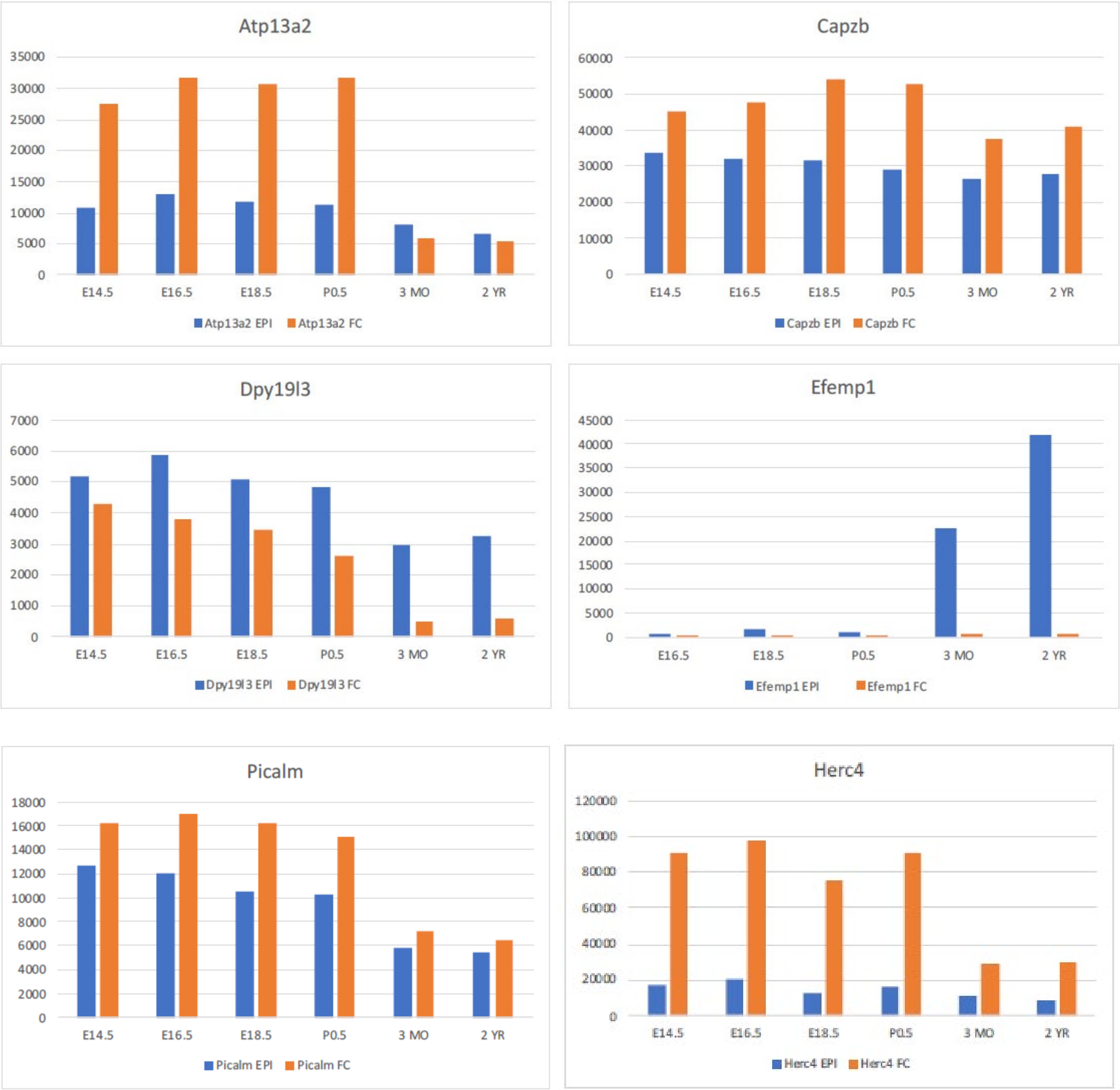

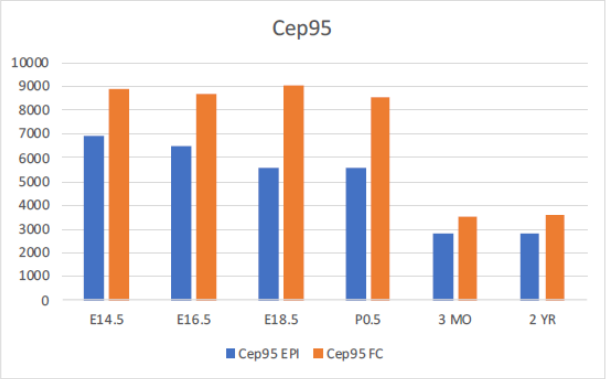
Cep95
10000
9000
8000
7000
6000
5000
4000
3000
2000
1000
0
E14.5
E16.5
E18.5
P0.5
3 MO
2 YR
Cep95 EPI
Cep95 FC

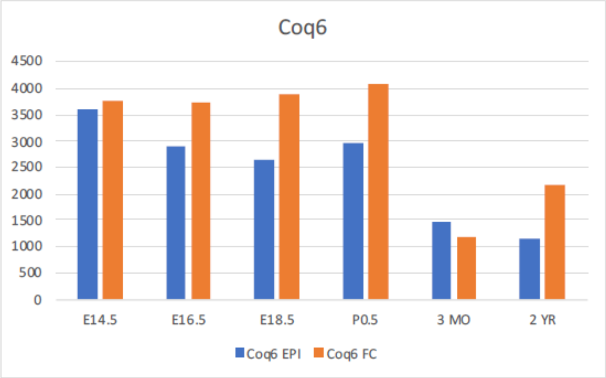
Coq6
4500
4000
3500
3000
2500
2000
1500
1000
500
0
E14.5
E16.5
E18.5
P0.5
3 MO
2 YR
Coq6 EPI
Coq6 FC

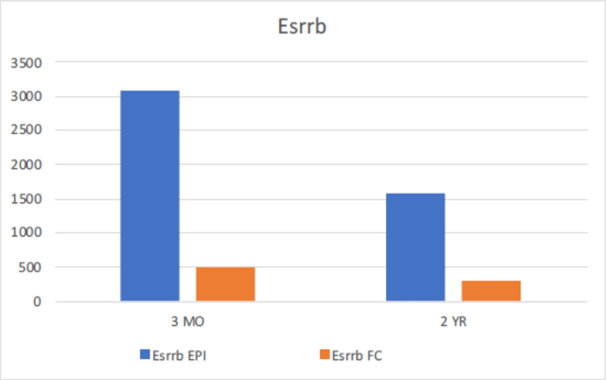
Esrrb
3500
3000
2500
2000
1500
1000
500
0
3 MO
2 YR
Esrrb EPI
Esrrb FC

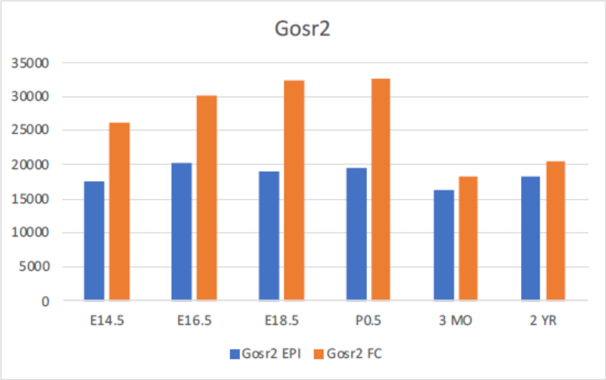
Gosr2
35000
30000
25000
20000
15000
10000
5000
0
E14.5
E16.5
E18.5
P0.5
3 MO
2 YR
Gosr2 EPI
Gosr2 FC

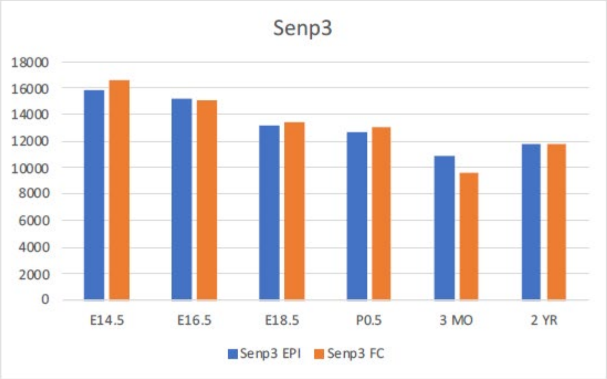
Senp3
18000
16000
14000
12000
10000
8000
6000
4000
2000
0
E14.5
E16.5
E18.5
P0.5
3 MO
2 YR
Senp3 EPI
Senp3 FC

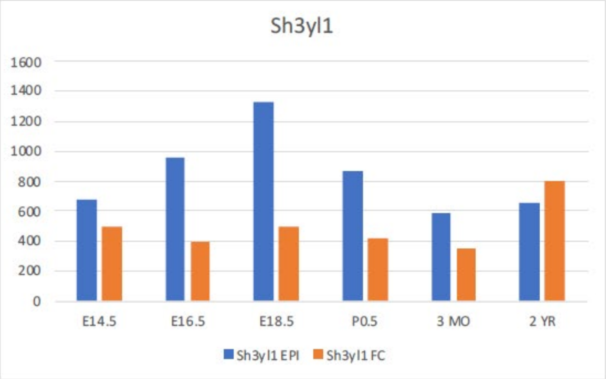
Sh3yl1
1600
1400
1200
1000
800
600
400
200
0
E14.5
E16.5
E18.5
P0.5
3 MO
2 YR
Sh3yl1 EPI
Sh3yl1 FC

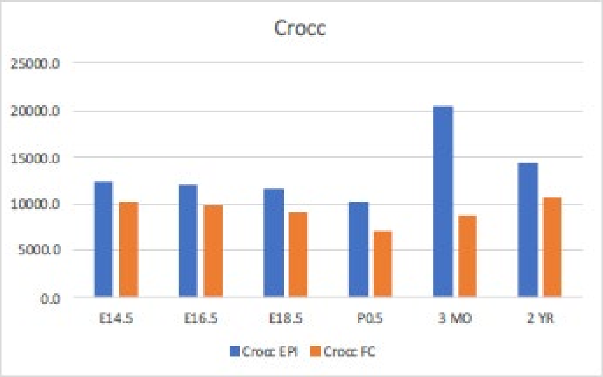
Crocc
25000.0
20000.0
15000.0
10000.0
5000.0
0.0
E14.5
E16.5
E18.5
P0.5
3 MO
2 YR
Crocc EPI
Crocc FC

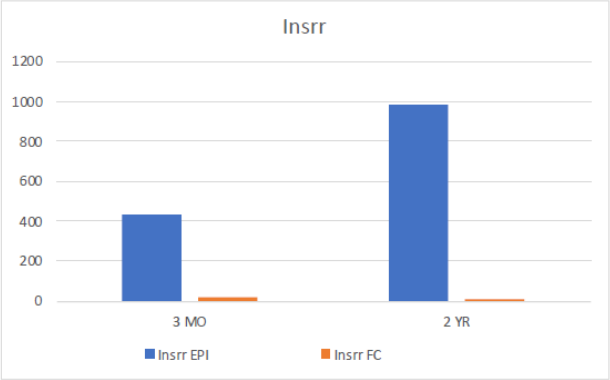
Insrr
1200
1000
800
600
400
200
0
3 MO
2 YR
Insrr EPI
Insrr FC

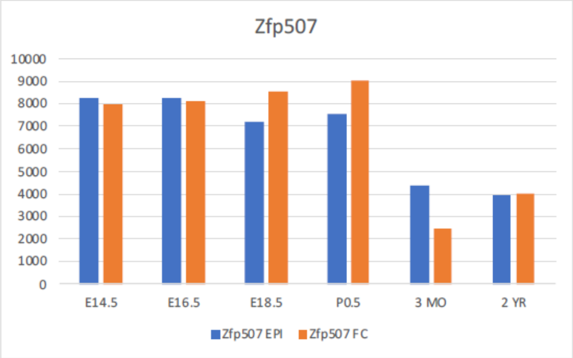
Zfp507
10000
9000
8000
7000
6000
5000
4000
3000
2000
1000
0
E14.5
E16.5
E18.5
P0.5
3 MO
2 YR
Zfp507 EPI
Zfp507 FC

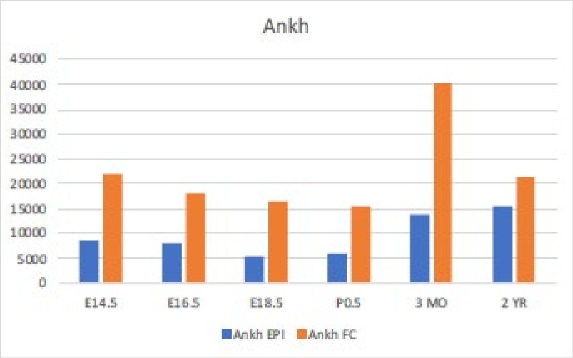
Ankh
45000
40000
35000
30000
25000
20000
15000
10000
5000
0
E14.5
E16.5
E18.5
P0.5
3 MO
2 YR
Ankh EPI
Ankh FC

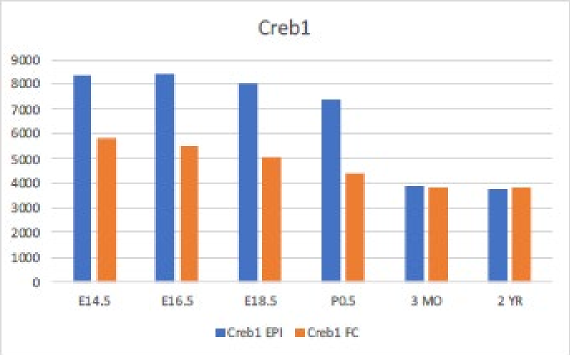
Creb1
9000
8000
7000
6000
5000
4000
3000
2000
1000
0
E14.5
E16.5
E18.5
P0.5
3 MO
2 YR
Creb1 EPI
Creb1 FC

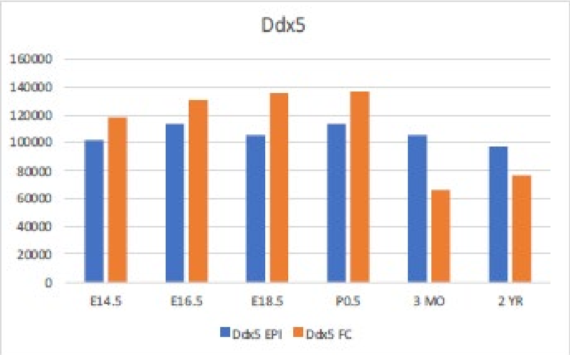
Ddx5
160000
140000
120000
100000
80000
60000
40000
20000
0
E14.5
E16.5
E18.5
P0.5
3 MO
2 YR
Ddx5 EPI
Ddx5 FC

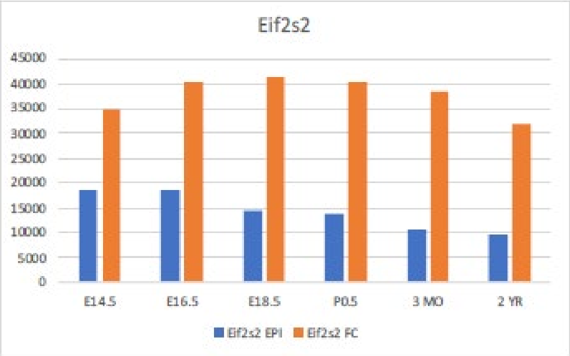
Eif2s2
45000
40000
35000
30000
25000
20000
15000
10000
5000
0
E14.5
E16.5
E18.5
P0.5
3 MO
2 YR
Eif2s2 EPI
Eif2s2 FC

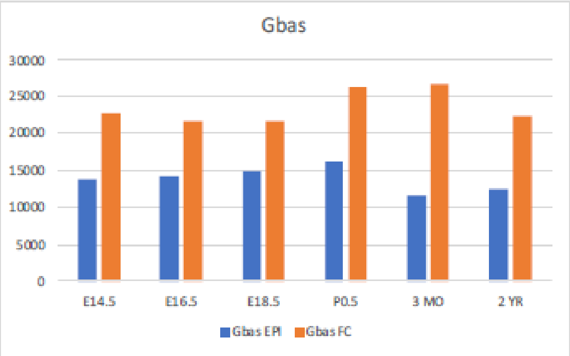
Gbas
30000
25000
20000
15000
10000
5000
0
E14.5
E16.5
E18.5
P0.5
3 MO
2 YR
Gbas EPI
Gbas FC

Supplementary Figure S6. Expression of novel cataract candidate genes is altered in various gene perturbation mouse models with lens defects. Examination of mouse orthologs of the human candidate genes for their expression in lens of different gene-perturbation mouse models of lens defects/cataract. Publicly available microarray data generated on the Affymetrix and Illumina platforms and meta-analyzed in iSyTE 2.0 were used to examine expression of mouse orthologs of human candidate genes in embryonic (E) or postnatal (P) stage lenses, ranging from mid lens development (*i.e.* E15.5) through adulthood (*i.e.* P56). Note: For Sparc, data at stage P28 represents expression in isolated lens epithelium. For Brg1 and Foxe3, the lens data is from overexpression models, while the lens data for other genes are from deficiency models. For individual candidate genes, significant alterations in lens expression is indicated by fold-change along with the associated *P* value for individual gene-perturbation mouse models at the specific age.

	E2f1,2,3 Mut E17.5	P Value	*E2f1,2,3* Mut P0	P Value	*HSF4* Mut P0	P Value	*Brg1* Mut E15.5	P Value
Ankh	-1.29	0.04	-1.29	0.05	-1.30	0.03	1.30	0.03

	E2f1,2,3 Mut E17.5	P Value	*E2f1,2,3* Mut P0	P Value	*Klf4* Mut P56	P Value	*Brg1* Mut E15.5	P Value
Atp13a2	-1.2	0.03	-1.21	0.03	-1.55	0.00	-1.86	0.00

	Tdrd7 Mut P30	P Value	*Klf4* Mut P56	P Value	*Brg1* Mut E15.5	P Value
Capzb1	1.28	0.01	-1.32	0.04	1.19	0.03

	E2f1,2,3 Mut P0	P Value	*Foxe3* Mut P2	P Value	*Brg1* Mut E15.5	P Value
Cep95	-1.28	0.01	-1.33	0.01	1.38	0.04

	Brg1 Mut E15.5	P Value
Creb1	-1.65	0.01

	E2f1,2,3 Mut P0	P Value	*Brg1* Mut E15.5	P Value
Crocc	-1.31	0.02	-1.71	0.00

	Foxe3 Mut P2	P Value
Coq6	-1.28	0.01

	Brg1 Mut E15.5	P Value
Ddx5	1.33	0.01

	E2f1,2,3 Mut E17.5	P Value	*E2f1,2,3* Mut P0	P Value	*Notch2* Mut E19.5	P Value	*Brg1* Mut E15.5	P Value
Efemp1	1.92	0.01	2.68	0.00	2.07	0.04	-1.66	0.04

	Brg1 Mut E15.5	P Value
Eif2s2	1.21	0.01

	Foxe3 Mut P2	P Value
Esrrb	-1.57	0.00

	Brg1 Mut E15.5	P Value
Gbas	-1.86	0.00

	Sparc Mut P28	P Value	*Brg1* Mut E15.5	P Value
Gosr2	-1.19	0.04	1.96	0.00

	Foxe3 Mut P2	P Value
Herc4	-1.38	0.00

	Notch2 Mut E19.5	P Value	*Foxe3* Mut P2	P Value
Insrr	-1.48	0.02	-1.45	0.01

	Notch2 Mut E19.5	P Value	*Brg1* Mut E15.5	P Value
Picalm	-1.33	0.04	1.36	0.00

	Klf4 Mut P56	P Value	*Sparc* Mut P28	P Value
Senp3	1.21	0.04	1.24	0.00

	E2f1,2,3 Mut P0	P Value
Zfp507	-1.33	0.00
