# Supplementary material for: Multi-tissue transcriptome-wide association study identifies novel candidate susceptibility genes for cataract
Source: Front Ophthalmol (Lausanne). 2024 Apr 16;4:1362350. doi: 10.3389/fopht.2024.1362350 (PMC11182099; doi:10.3389/fopht.2024.1362350)
Supplement: Supplementary file 1 [file DataSheet_1.pdf]

DIVISION OF RESEARCH
Northern California

Kaiser Permanente
Research

To the *Frontiers in Ophthalmology* Editor:
Barbara Pierscionek - Associate Editor

March 22, 2023

Dear Dr. Pierscionek,

We would like to thank you for your interest in our manuscript entitled "**Multi-tissue transcriptome-wide association study identifies novel candidate susceptibility genes for cataract**" (Manuscript ID: 1362350). We also thank the two Reviewers for their very enthusiastic feedback and for their helpful comments and suggestions on the manuscript. We have revised the manuscript to address the individual comments and hope that it is now acceptable for publication.

As recommended by Reviewer #1, we added some discussion about the possibility of co-regulation between the genes identified within the 11q13.3 genomic region. As suggested by Reviewer #2, we added a flowchart (that summarizes the utilized data sources, TWAS analyses and results) in the Supplementary Material (Supplementary Figure S1). As raised by Reviewer #2, we revised the language of the current article to prevent repetition in the description of the Methods between the current TWAS study of cataract and our previous TWAS study of migraine (Meyers et al. HGG Adv. 2023). Finally, we emphasized the different methods, software version, and data sources used between the two TWAS studies.

We provide detailed responses addressing the individual points raised by the two Reviewers in a separate document. We also provide a marked-up version of the revised manuscript with the changes highlighted in yellow.

We confirm that this manuscript has not been published elsewhere and is not under consideration by another journal. The authors declare no conflict of interest. We look forward to its publication in *Frontiers in Ophthalmology* for the upcoming article collection "*Women in Lens and Cataract*".

Sincerely,

Hélène Choquet, Ph.D.
Research Scientist II (Associate Professor Equivalent)
Kaiser Permanente, Division of Research
2000 Broadway - Oakland, CA 94612
