# Supplementary material for: Multi-tissue transcriptome-wide association study identifies novel candidate susceptibility genes for cataract
Source: Front Ophthalmol (Lausanne). 2024 Apr 16;4:1362350. doi: 10.3389/fopht.2024.1362350 (PMC11182099; doi:10.3389/fopht.2024.1362350)
Supplement: Supplementary file 3 [file DataSheet_3.pdf]

**Independent Review Report, Reviewer 1**

This manuscript reports results from a transcriptome-wide association study (TWAS) of cataract. The study identified 86 genes associated with cataract, and 15 of them are not overlapping with previous GWAS hits. The study also identified that gene expression is not only associated with lens but associated with other tissues, especially gastrointestinal tissues. The study suggests that the TWAS is a useful method to identify new loci associated with cataract as identified in other diseases previously. It also shows that it may be useful in identifying how associated (both previously identified and novel) loci affect gene expression. Identification of a gene-gastrointestinal association specifically suggests a broader, systemic contribution to cataract development, beyond the traditionally focused lens tissue. This insight opens new avenues for understanding the pathophysiology of cataracts and potentially other diseases, by considering the role of extra-lenticular tissues.

The methodology adopted in the study is robust, and the discussion thoughtfully addresses the limitations of the approach, providing a balanced view of the findings. However, to enhance the manuscript further, a couple of points warrant additional consideration:

We thank the reviewer for the positive feedback and the constructive review.

**1)** Nine genes are located within the 11q13.3 genomic region which was previously identified. Some of the genes show positive association while others show negative association. It would be nice to analyze this genomic region a little deeper. Do all of the genes potentially contribute to the phenotype or do some of them stand out by bioinformatic analysis? How are positively correlated genes or negatively correlated genes potentially regulated? Are they individually regulated or is there any sequence that affects expression of multiple genes within the region? Further analysis and discussion would benefit to understand the etiology of previously identified loci, which could be another benefit for the TWAS analysis.

Thanks for raising this important point. We would like to specify that among the 202 gene-tissue pairs identified in our tissue-specific TWAS analysis, 166 (82.2%) correspond to 9 unique genes (i.e., *TESMIN*, *AP000808.1*, *MRPL21*, *IGHMBP2*, *MRGPRD*, *MRGPRF*, *AP003071.4*, *MRGPRF-AS1*, and *TPCN2*) that are located at the previously reported 11q13.3 cataract-associated locus (Choquet et al. Nat Comms 2021). Those 166 gene-tissue pairs reached the Bonferroni significance threshold for their associations between imputed gene expression with cataract, suggesting that all of the 9 genes contribute to the disease susceptibility. While increased predicted expression was associated with cataract risk for 68 gene-tissue pairs (i.e., TWAS z-score > 5.0), decreased predicted expression was associated with cataract risk for 98 gene-tissue pairs (i.e., TWAS z-score < -5.0).

We would like to emphasize that, despite the great success in prioritizing gene-trait associations in complex diseases and traits, TWAS may present multiple hits per locus, owing to co-regulation, which remain problematic (PMIDs: 30926968 and 37658226). We have added some text and related references in the Discussion to reflect this potential limitation, as follows: "*Our study should be interpreted within the context of its limitations. (…) Despite the great success in prioritizing gene-trait associations in complex diseases and traits, TWAS may present multiple hits per locus, owing to co-regulation, which remain problematic. Thus, future models could consider more complex genetic architecture containing different regulatory effects and our TWAS results could benefit from subsequent functional assays to indicate the potential targets underlying those associations, notably at 11q13.3*."

**2)** "been linked to eye diseases and…." In the discussion

The discussion section mentions the linkage of certain findings to eye diseases without specifying the types of eye diseases or providing references. For clarity, it would be beneficial to specify the eye diseases referenced and include supporting literature.

We would like to clarify that, to date, there are no mutations in either *INSRR* or *CEP95* that have been linked to eye diseases. We have now rephrased this sentence in the Discussion, as follows:

"*To date, no mutations in either INSRR or CEP95 have been linked to eye diseases, and further studies are needed to confirm the role of these genes in cataract etiology and determine their precise role in cataract susceptibility*."

**Independent Review Report, Reviewer 2**

This study presents interesting findings from tissue-specific and multi-tissue TWAS analyses, unveiling 86 genes associated with cataract. Notably, 15 of these genes are novel and not identified in previous GWAS analyses, thus enriching the knowledge base for researchers studying cataract.

We thank the reviewer for the positive feedback and the helpful comments.

**Minor Points:**

To enhance clarity, incorporating a flow chart illustrating the utilized datasets and analyses would provide valuable guidance for readers, considering the extensive use of various datasets and analyses methods.

We have now added a flowchart (that summarizes the utilized data sources, TWAS analyses and results) in the Supplementary Material (as Supplementary Figure S1).

Furthermore, while similar of their previous work on migraine (Meyers et al., 2023), authors should consider revising the language in this article to prevent repetition and maintain its distinctiveness.

We carefully revised the language of the current article to prevent repetition in the description of the Methods between our previous TWAS study of migraine (Meyers et al. HGG Adv. 2023) and the current TWAS study of cataract. Also, we would like to emphasize that while we utilized the same TWAS approach (i.e. FUSION) to conduct the 2 studies, for the current study, we used the most recent version of FUSION software (version 8) that implemented the Genotype Tissue Expression (GTEx) Project v8 models for 49 GTEx tissues (in contrast to our previous TWAS of migraine that used FUSION v7 that implemented the GTEx Project v7 models for only 48 GTEx tissues). In addition, in the current TWAS study, we examined the expression of the novel identified cataract genes in lens tissues using the iSyTE database (we did not conduct such analyses in our previous TWAS of migraine).
